# Multi-Laboratory Assessment Reveals Variable Ion Species Profiles in Electrospray Ionization Mass Spectrometry

**DOI:** 10.1021/jasms.6c00011

**Published:** 2026-05-06

**Authors:** Melanie T. Odenkirk, Jacqueline M. Chaparro, Nathan T. Montgomery, Katrina L. Leaptrot, Stacy D. Sherrod, Jody C. May, Henriëtte van Eekelen, Bert Schipper, Juliana Chaura, Gabriel Esteban Velez, Wimonphan Chathiran, Cole R. Michel, Katrina A. Doenges, Richard Reisdorph, Mari Maeda-Yamamoto, Luis Valdiviez, Uri Keshet, Jeremiah Wells, Scott Mackell, David Bell, Sebastian Tapia Leiva, Simon Partridge, Daniela Rago, Linda Ahonen, Yuki Ito, Margaret L. Read, John C. Evans, Macy J. Gruszczynski, Leokadja Szalach, Stephen C. Boyko, Steven Watkins, Rodrigo Ledesma Amaro, Oliver Fiehn, Nichole Reisdorph, Warangkana Srichamnong, Andres Jaramillo-Botero, Ric C. H. de Vos, Robert D. Hall, John A. McLean, Corey D. Broeckling, Jessica E. Prenni

**Affiliations:** † Department of Horticulture and Landscape Architecture, 3447 Colorado State University, Fort Collins, Colorado 80521, United States; ‡ Analytical Resources Core, Colorado State University, Fort Collins, Colorado 80521, United States; § Department of Chemistry and Center for Innovative Technology, 5718 Vanderbilt University, Nashville, Tennessee 37240, United States; ∥ iÓMICAS Research Institute, 27965 Pontificia Universidad Javeriana Cali 760031, Colombia; ⊥ Business Unit Bioscience, 4508 Wageningen University & Research, Wageningen 6708 PB, The Netherlands; # Institute of Nutrition, 26685 Mahidol University, Nakhonpathom 73170, Thailand; ¶ Skaggs School of Pharmacy and Pharmaceutical Sciences, 129263 University of Colorado, Aurora, Colorado 80045, United States; ∇ Institute of Food Research, National Agriculture and Food Research Organization (NARO), Tsukuba Ibaraki 305-8642, Japan; ○ West Coast Metabolomics Center, Davis, California 95616, United States; ⧫ Department of Infectious Diseases, Faculty of Medicine, 4615 Imperial College London, London SW7 2AZ, U.K.; †† Novo Nordisk Foundation Center for Biosustainability, 587234 Danmarks Tekniske Universitet, Kongens Lyngby 2800, Denmark; ‡‡ 65828 Shimadzu Corporation, Kyoto 604-8511, Japan; §§ Verso Biosciences, Davis, California 95618, United States

**Keywords:** electrospray (ESI), ionization, mass spectrometry (MS), metabolomics, molecule

## Abstract

Electrospray ionization (ESI) is a foundational technique in mass spectrometry (MS) widely applied to measure nonvolatile molecules in diverse chemical and biological samples. Yet variation in ESI-MS instrumentation, operating settings, and parameters such as solvent composition creates specific, local ionization environments that drive the formation of select ion species for individual analytes. As ESI continues to advance analytical discovery, understanding the extent to which variation in ion species formation impacts intra- and inter-experimental results is essential. Here, we assessed ion species formation by analyzing an internal retention time standard (IRTS) mixture across ten laboratories employing high-resolution ESI-MS instrumentation from four vendors (Agilent Technologies, ThermoFisher Scientific, Shimadzu Corporation, and Waters Corporation). Instrument vendors were considered not as a benchmark of performance, but as a practical framework to capture differences in source design, ion optics, and analyzer/detectors that are inherently coupled to commercial platforms. Despite the use of standardized extraction and chromatographic protocols, differences in instrument configuration, source conditions, and method execution resulted in variation in ion species formation across vendors, among laboratories using instruments from the same vendor, and even within individual laboratories. These findings demonstrate that, even with standardized methods, the collective influence of local ionization environments on ion species formation remains a critical obstacle for interpreting LC–MS small molecule data and improving reproducibility and comparability across studies.

## Graphical abstract

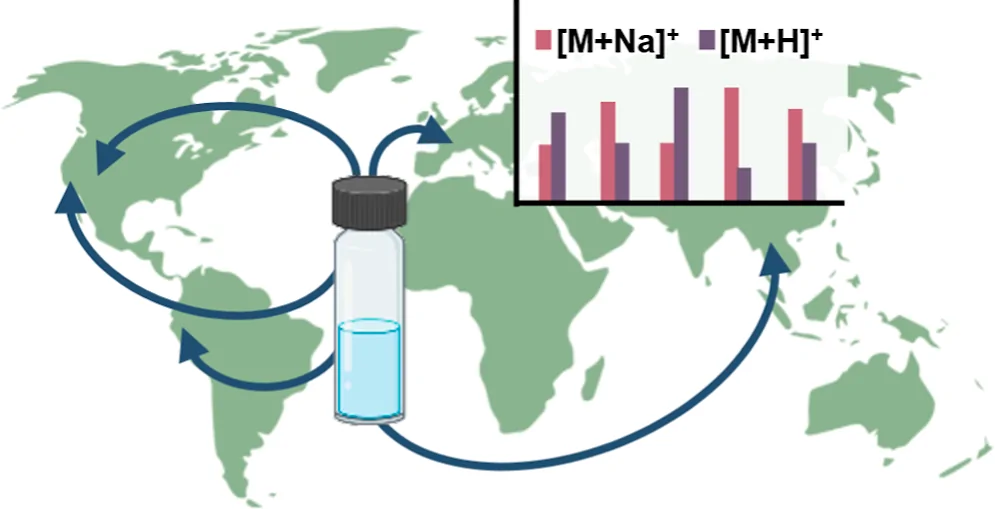

## Introduction

Since its initial publication in 1984, electrospray ionization (ESI) has had a transformative impact on mass spectrometry by enabling the ionization of nonvolatile molecules.^1,2^ This advancement has catalyzed a wide range of research initiatives, including proteomics, metabolomics, and lipidomics.^3–5^ Within proteins, ESI has deepened our understanding of protein expression, configuration, and modification, elucidating biological signaling mechanisms and their perturbation across a variety of disease states.^6,7^ Similarly, metabolomics and lipidomics have leveraged ESI-MS to characterize plant response to environmental stressors, disease pathogenesis in humans, and the complex interactions between agriculture, food and human health.^8–11^ Technological innovations alongside ESI have also enabled the measurement of macromolecules, including kilodalton- to megadalton-scale systems such as native membrane proteins, RNA-ligand complexes, and viral capsids.^12–14^

As a result, ESI is now a widely commercialized ionization technique. Although MS vendors apply the same core principles, the engineering of source geometries and auxiliary mechanisms to facilitate ion formation (e.g., sheath/curtain gases and elevated temperatures) varies across platforms and applications.^15^ For example, Agilent Technologies Jet Stream ESI technology uses a heated sheath gas to enhance ion transmission.^16^ Conversely, ThermoFisher’s heated ESI (HESI) source, implemented on select Orbitrap instruments, uses a heated sheath gas that is diffusely applied along the capillary, a notable difference to the focused application of the Jet Stream systems.^17^ On other ESI-MS systems, such as Shimadzu instrumentation, dual ionization capabilities have been engineered to allow for fast switching between ESI and atmospheric pressure chemical ionization (APCI) to further expand the measurable chemical space for small molecules.^18^ In practice, this design often requires a trade-off of reduced ESI capillary voltage that is compensated by an auxiliary heater in ESI applications. Postionization, commercial MS instrumentation also use unique ion paths and focusing techniques to facilitate downstream analysis and detection.^19^ As each vendor has refined ESI and ion transfer processes on their systems, evidence has shown that variations in source design and local operating conditions across vendors and through evolving instrumentation from the same vendor influence ion formation.^20–23^

However, ionization is not only influenced by source design and operation. Namely, the chemical composition of the spray solution within the ESI microenvironment is a key modifiable parameter that can be curated to drive specific ion formation pathways.^24–26^ For example, solvent composition strongly influences droplet behavior, with more organic solvents promoting faster desolvation, while protic solvents stabilize hydrogen-binding clusters and promote the formation of protonated [M + H]^+^ adducts.^18,27^ Additionally, acid/base additives and salt modifiers are also widely used to enhance ionization efficiency and direct adduct formation. For example, ammonium salts can promote [M + NH_4_]^+^ formation, while acids and bases (e.g., formic acid or ammonium hydroxide) can shift the protonation equilibrium, thereby biasing ionization toward specific adduct species.^20,25,28,29^ In summary, ESI is a powerful and variable ionization process influenced by both controllable and uncontrollable factors.

At a fundamental level, factors influencing the local ESI ionization environment have substantial effects on ion species formation. Combined with an incomplete understanding of ESI mechanisms, this leads to multifactorial variability that challenges the reproducibility and comparability of data generated by ESI-MS.^25,26,30,31^ A critical aspect of this variability is ion species formation (including adducts, neutral losses, and multimers), a pivotal step in ion generation, detection, annotation, and quantification.^26,32^ Although the importance of ion species formation in ESI-MS has long been acknowledged, there has been limited systematic evaluation of how the patterns of ion species formation diverge in real-world applications, where instrument platforms and methods implementation may vary. This gap is particularly pronounced for the diverse landscape of small molecules and understanding how ion species formation affects both targeted and nontargeted metabolomics applications.

Herein, we present critical insights into the variability of ion species formation by leveraging a custom internal retention time standard (IRTS)^33^ mixture across ten laboratories following the same standardized nontargeted metabolomics protocol developed by the Periodic Table of Food Initiative (PTFI).^33^ All laboratories were equipped with high resolution mass spectrometry instrumentation from multiple vendors, including Waters (one lab, W1), Thermo (three laboratories, T1-T3), Shimadzu (two laboratories, S1 and S2), and Agilent (four laboratories, A1–A4). Importantly, extraction and chromatographic methods were standardized or otherwise documented to capture variation in method implementation.^33^ MS settings, including electrospray ionization, were optimized by each laboratory. Collectively, the data collected provides insights into how method implementation factors affect real world execution of a standardized method, influencing ion species formation and, in turn, small molecule identification and quantification.

## Methods

### Materials and Reagents

#### Internal Retention Time Standard

The reversed-phase internal retention time standard (IRTS) was prepared by AnalytiCon Discovery (Postdam, Germany). The 33 compounds included in the IRTS mixture were selected for their (i) detection by LC-ESI-MS, (ii) unlikeliness to be naturally occurring in food, (iii) separation across the full chromatographic range of a standardized reverse phase method, and (iv) scalability for eventual commercialization into a globally distributable reagent for nontargeted metabolomics.^33^ A full list of the IRTS compounds and their physicochemical properties computed using rdkit (version 2025.09.3) is presented in Supporting Information Table S-1.^34^

### Carrot Procurement and Processing

Orange carrots (Daucus carota subsp. sativus) were purchased from a supermarket in Fort Collins, Colorado, USA. A total of approximately 200 g of commercially available carrots were randomly selected and transported to the laboratory under standard commercial handling and storage conditions. All carrots were combined to generate a single composite specimen that was processed as a collective sum.

Following procurement, carrot samples were prepared by removing the tops and outer skin, chopped into half-inch pieces, and frozen at −80 °C. The frozen carrot pieces were then lyophilized using a Labconco lyophilizer before reaching complete dryness after 72 h. Dried carrot pieces were homogenized using a coffee grinder and divided into 55 ± 5 mg aliquots. These samples were stored at −80 °C until distribution, where samples were shipped alongside dried IRTS material on dry ice to participating laboratories. Laboratories A2 and T1 procured and processed local orange carrots independently using similar methodologies (Table S-2).

### Lab-Specific Reagents

Laboratories received IRTS and carrot samples as dried, powdered materials, which were stored at −80 °C upon receipt. Each laboratory performed resuspensions, extraction, and LC–ESI-MS data acquisitions following a standardized protocol that allowed for flexible selection of reagents and materials. All solvents were procured by vendors preferentially selected by each laboratory to reflect existing resources for laboratories across global locations.^35^ A full list of the lab-specific reagents used is presented in Supporting Information Table S-3.

### Sample Preparation

#### IRTS Resuspension and Dilutions

Once received, IRTS samples were resuspended in 1.1 mL of an 80:20 methanol/water (MeOH/H_2_O) solution prepared by each laboratory. For laboratories A2 and T1, this solution was used to prepare IRTS solutions at a variety of concentrations. All other laboratories prepared a single-concentration IRTS solution, which was then extracted via solid phase extraction (SPE) as described below.

### Solid Phase Extraction

Carrot and blank IRTS samples were extracted following the PTFI standardized nontargeted metabolomics method.^33^ Agilent SPE Captiva Cartridges were first conditioned with a 120 μL addition of 80:20 MeOH/H_2_O and 480 μL of a 5:1 acetonitrile/water solution with 1% formic acid (FA) (crash solution). Laboratories W1, A1, A4, S2, and T2 implemented a positive pressure manifold to dispense solvents at 1 drop every 3–5 s. Laboratories T1, T3, A2, A3, and S1 used a complementary vacuum filtration system to achieve similar elution of solvent through SPE cartridges. Once complete, samples were left under gas flow for an additional 10 min to ensure complete dryness.

Lyophilized carrot samples were allowed to reach room temperature (∼30 min) before resuspending in 12 μL of 80:20 MeOH/H_2_O for every 1 mg of food. Laboratories also created IRTS extraction blanks at this step by adding 600 μL of 80:20 MeOH/H_2_O to empty tubes. Samples were vortexed for 10 min before centrifuging at 14,000 RCF. Both steps were completed at room temperature. Supernatant (120 μL) of each sample was then introduced to the SPE cartridges, which were preloaded with 480 μL of crash solution. An additional 10 μL of resuspended IRTS was added to each sample before pressurizing systems under nitrogen, where again, the flow was set to one drop every 3–5 s.

The collected eluent was then centrifuged for 10 min at 3500 RCF before drying. For all laboratories except A3 and T2 which used a SpeedVac, drying was completed with a nitrogen evaporator. Samples were then resuspended in 80:20 MeOH/H_2_O to an optimal volume that had previously been determined for their instrument (data not shown). Samples underwent a final 10 min vortex at room temperature before being temporarily stored until LC–MS analysis. Specific parameters for extraction conditions are presented in Supporting Information Table S-4, with solvents presented in Table S-3.

## Data Collection

### LC–MS Data Acquisition

Reversed-phase LC–ESI–MS data were collected by each laboratory following the PTFI’s standardized nontargeted metabolomics workflow.^33^ Chromatographic separations were completed using an Agilent Zorbax SB-Aq RRHD column (2.1 mm × 100 mm × 1.8 μm particle size) with an Agilent in-line filter (0.3 μm, 2 mm I.D.) held at 30 °C. Mobile phase A consisted of LC–MS grade water (H_2_O) with 0.1% LC–MS grade FA while mobile phase B was LC–MS grade ACN with 0.1% LC–MS grade FA. All laboratories implemented the same chromatographic separation with the solvents presented in Table S-3. The flow rate was set to 0.6 mL/min across a 15 min linear gradient. The LC method began at 1.8% B. After maintaining this composition for 1 min, B ramped to 90% for an additional 10 min. These conditions were held for 1.8 min before re-equilibrating the column to 1.8% B for the remaining 3.2 min. Two μL of samples were loaded onto the column for separation by all laboratories except A4. Here, the addition of IMS separations required a 4 μL injection of sample and 4-bit IM multiplexing to enhance signal intensity.^36,37^

Mass spectrometry data were collected in precursor-only mode across the one Waters, two Shimadzu, three ThermoFisher, and four Agilent instruments included in this study. For TOF analyzers, the acquisition rate was 2–3 scans per second. Additionally, it was recommended that laboratories collect data in centroid mode to support file sharing. Other mass spectrometer settings were left as discretionary considerations, with guidance to optimize individual systems for small molecule analysis. Specific parameters and model information for each laboratory are presented within each vendor in Supporting Information Tables S-5–S-8.

### Confirmatory IMS Data Acquisition

LC–IM–MS data were collected by the Center for Innovative Technology at Vanderbilt University (Lab A4) on an Agilent 1290 Infinity II UHPLC coupled to an Agilent 6560 IM-QTOF MS platform. IM-MS data were collected with both positive and negative ESI from *m*/*z* 50 to 1700 at a cycle time of 2.1 frames per second. Following ESI, ions were accumulated in a trap for 3900 μs (μs) before being released for 180 μs into an ion mobility drift tube operated with ultrahigh purity (UHP) nitrogen. Ion mobility scans were collected using a transient rate of 7 with 4-bit multiplexing to enhance the duty cycle. The max drift time for the separation was set to 60 ms.

### Confirmatory DDA Data Acquisition

To validate select IRTS adducts of species, data dependent acquisition (DDA) data were collected by the Analytical Resources Core at Colorado State University (Lab W1) using the same LC and ESI settings as presented in Supporting Information Table S-4. Waters DDA data were collected on the Xevo MRT using an inclusion list to preferentially select adducts for individual IRTS molecules (Table S-9). Dynamic exclusion was also used with an exclusion window of 6 s and a 100 ppm mass difference. Precursor scanning was initially performed across *m*/*z* 50–1200 at a rate of 0.1 s/scan. Acquisition switched to MS/MS when individual ion intensity exceeded 3000. Here, MS/MS data were acquired until ion accumulation reached a TIC threshold of 100,000 or 0.2 s of data were acquired. Throughout MS/MS acquisition, LM resolution was set to 6.0 to limit the quadrupole transmission window to the M0 isotope.

### Data Analysis

Raw, vendor-encoded LC–ESI–MS data were processed using Skyline (Version 24.1.0.414).^38^ Target libraries were developed for IRTS molecules observed in either negative or positive mode. Positive ESI ion species searched included protonated ([M + H]^+^), sodiated ([M + Na]^+^), potassiated ([M + K]^+^), ammonium adduct ([M + NH_4_]^+^), and protonated dimer ([2M + H]^+^) forms. For negative ESI, ion species searched included deprotonated ([M – H]^−^), formate adduct ([M + COOH]^−^), acetate adduct ([M + C_2_O_3_H]^−^), chlorine adduct ([M + Cl]^−^), and deprotonated water loss ([M – H_2_O – H]^−^).

Data across instruments were processed together by ionization mode. Candidate peaks for each IRTS were evaluated based on their mass accuracy (≤5 ppm) and proximity of raw retention time values to previously established PTFI retention time library values.^33,39^ Notably, the PTFI retention time library had variable accuracy across instrumentation, as configuration differences such as dead volume from the LC outlet to the MS inlet are unaccounted for in raw retention time data.^40^ However, the consistency of these differences resulted in reproducible early or late elution of peaks that still supported the identification of IRTS regardless of these differences. Once identified, all IRTS signals were manually integrated prior to the export of final data (Table S-10). Transitions for all data as well as raw, vendor-encoded files are available on Panorama Public (https://panoramaweb.org/5co3dQ.url).^41^

### IMS Data Analysis

LC–IM–MS data were processed using the PNNL PreProcessor (Version 4.0) to deconvolute the multiplexed signal.^42^ Files were then uploaded to IM-MS Browser (Agilent, Version 10.0), where they underwent alignment using calibrant ions (tune mix) acquired at the beginning of experiments.^43^ From there, IRTS adducts were searched, and the arrival times and experimental collisional cross section (CCS) values were determined using the single-field CCS method (eq 1).^43^

1 $$CCS=\left(t_{A}-t_{fix}\right)\frac{z}{\beta }\sqrt{\frac{m_{B}+m_{a}}{m_{a}}}$$

Here, *t*_A_ and *m*_a_ are the arrival time and mass for a particular ion species, *m*_B_ is the buffer gas (N_2_) mass, and *z* is the ion charge. The parameters β and *t*_fix_ are obtained from the calibration curve slope and *y*-intercept as determined from the regression of measured drift times and standard CCS values of the tune mix. Experimental CCS values are presented in Supporting Information Table S-11.

### DDA Data Analysis

DDA data were analyzed using SeeMS software, where precursor molecules were selected based on their *m*/*z* and retention time values recorded from previous acquisitions.^44^ Manual processing was performed where the elution window was isolated to extract DDA data for each target analyte. Data was acquired for both an IRTS sample and a blank of 80:20 MeOH/H_2_O. Spectral data was then input into R (Version 4.4.3) for plotting Raw DDA spectra in IRTS and solvent blank samples using ggplot2 (version 4.0.0) and dplyr packages (version 1.1.4).^45^ Raw data is also available on the Panorama Public page (https://panoramaweb.org/5co3dQ.url).^41^

### Data Processing, Visualization and Statistical Analysis

#### Data Processing

Exports from Skyline were processed in R (Version 4.4.3) using tidyverse packages (version 2.0.0).^45^ This included an initial merge of extracted ion chromatogram (EIC) data with metadata to define laboratory, instrument, and sample type for each injection. Intensity (peak area) cutoffs of 2000 and 5000 were used for negative mode and positive mode, respectively. These thresholds were determined by computing average noise values and multiplying by three. To remove false annotations, an additional mass filter of ≥15 ppm was used. Any annotations that did not align with the PTFI retention time library values for the IRTS compounds (±1 min) were manually removed from the data before proceeding to data alignment.

To account for the variable resuspension volumes (RV) and injection volumes (IV) optimized for each laboratory, a dilution factor (DF) was defined. This term also included a sample concentration factor (SCF) to account for double-concentrated (2X) samples, where appropriate (eq 2).

2 $$DF=\frac{RV\left(\mu L\right)/IV\left(\mu L\right)}{SCF}$$

This dilution factor was multiplied with the raw area values to account for variability in IRTS preparation and to ensure optimal analysis on each instrument. This data was then normalized to the total IRTS signal of all ions within each injection to complete quantitative alignment of data (Table S-12).

### Ion Species Formation Preferences

The processed data (Table S-12) were then used to qualitatively assess ion species formation across laboratories and among replicate injections within a laboratory. For each IRTS compound in each injection, the most abundant ion species was first identified. To evaluate differences between laboratories, the most abundant ion species were aggregated through a count across laboratories. The same approach was applied to replicate injections to evaluate consistency within instrumentation for comparisons within the same vendor and within individual laboratories. All analyses were performed separately for blank + IRTS and carrot + IRTS datasets.

To evaluate profiles, box and whisker plots of the observed ion species and their relative intensities were generated to compare relative adduct species distributions across platforms. Statistical analysis was performed using a Kruskal–Wallis test followed by a Dunn’s test to determine pairwise significance.^46,47^ All *p*-values were adjusted using the Benjamini–Hochberg correction before determining significant changes (α ≤ 0.05).^48^ Outputs from these tests are presented in Supporting Information Table S-13. Within-lab ion species profiles were assessed by comparing individual replicates collected for IRTS + blank or IRTS + carrot samples.

## Results and Discussion

To evaluate differences in ion species formation, we implemented the Periodic Table of Food Initiative (PTFI) standardized nontargeted metabolomics method across ten laboratories (Figure 1A). For analysis, all laboratories were sent the PTFI internal retention time standard (IRTS) mixture that was developed to support comparability of this method by aligning chromatographic data (see Supporting Information Table S-1 for full IRTS list). The IRTS mix was shipped as a dried powder, ensuring consistent sample integrity for the global network of participating laboratories. Upon receipt, laboratories implemented a standardized workflow for resuspending and preparing the IRTS before acquisition of LC–MS1 data. In execution, this resulted in some variation in method translation due to differences in available equipment and reagents. However, all materials were noted and required to meet minimum standards (i.e., all solvents being LC–MS grade reagents) to account for potential effects on ion species formation. Examples of this include solvent suppliers for items like LC–MS grade acetonitrile (ACN) varying based on feasibility of procurement, cost, and lab preferences (see Table S-3 for more solvent information). Similarly, sample preparation using solid phase extraction (SPE) was completed using either a positive pressure manifold or vacuum filtration based on available equipment. Once prepared for LC–MS1 analysis, laboratories implemented the standardized chromatographic method on instrumentation that was independently optimized for acquisition of small molecule data in positive and negative ESI.

**Figure 1:**
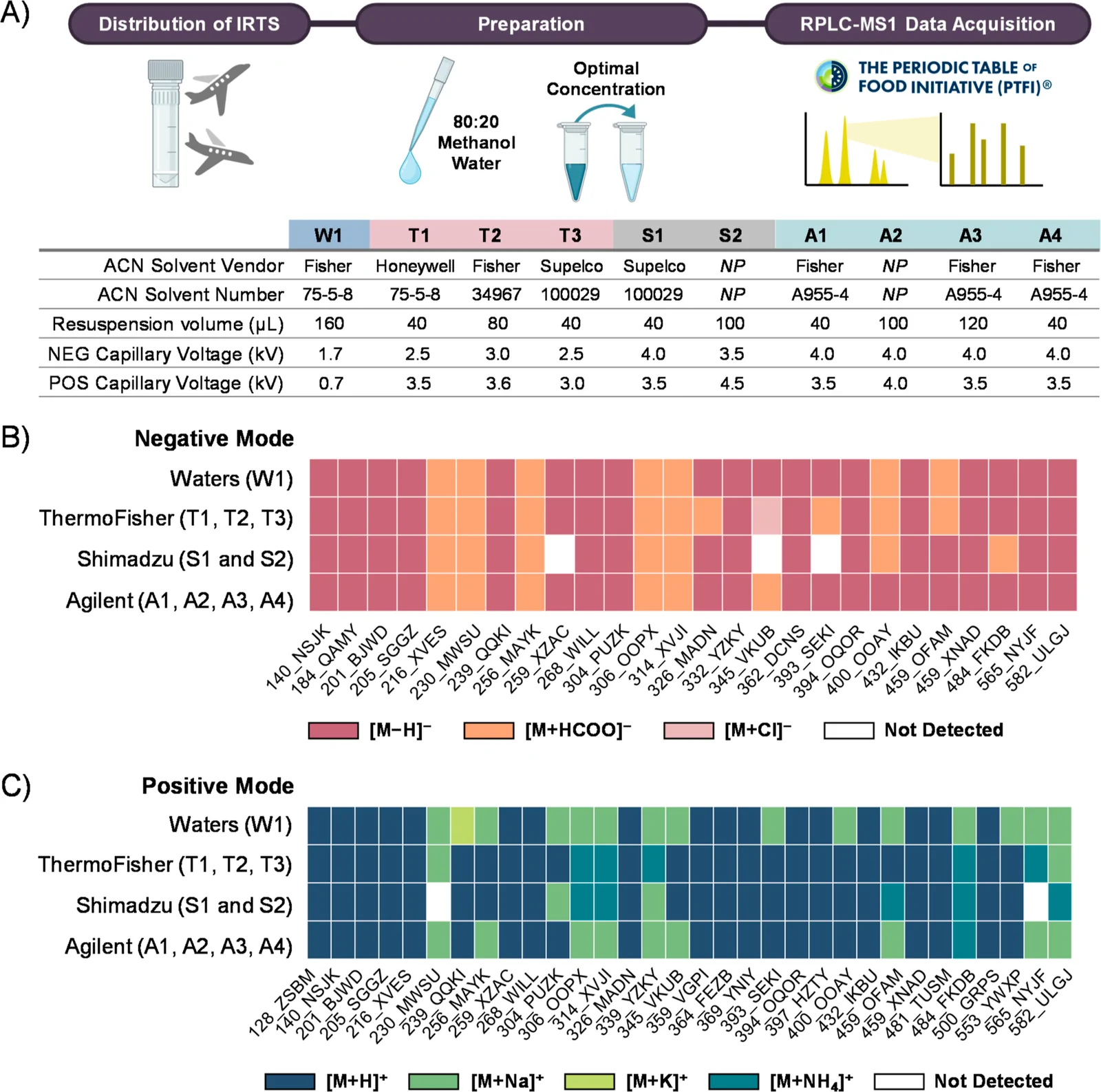
Overview of ion species formation for all IRTS compounds across vendors. (A) Schematic of experimental setup, where a homogeneous IRTS mixture was distributed internationally to partner laboratories. Laboratories first prepared dried standard material by resuspending it in 80:20 methanol/water and then diluting the sample to the optimal concentration (data not shown). Laboratories collected data following the PTFI nontargeted metabolomics standardized reversed phase LC method.^33^ MS data were collected using parameters optimized for small molecule analysis by each laboratory. Despite standardized protocols, convenience and logistics resulted in variations of method adoption across laboratories, including sources of solvents, resuspension volumes, and instrumentation settings. (B) The most abundant negative mode ion species observed for each IRTS compound is depicted across all instrument vendors ([M – H]^−^, red; [M + HCOO]^−^, orange; [M + Cl]^−^, pink; and undetected, white). (C) The most abundant positive mode ion species of each IRTS compound across all instrument vendors ([M + H]^+^, blue; [M + Na]^+^, green; [M + K]^+^, lime green; [M + NH_4_]^+^, turquoise; and undetected, white). For both graphs, the most abundant ion species was determined by counting the most abundant ion species across all injections. A complete reference of IRTS compounds and method implementation and instrumentation parameters are available in Supporting Information Tables S-1, S-3–S-8.

The results presented here reflect data collected across 10 laboratories, each with unique instrument models from four leading commercial vendors: Agilent Technologies (4 laboratories, A1-A4), Shimadzu Corporation (2 laboratories, S1–S2), Thermofisher Scientific (3 laboratories, T1–T3), and Waters Corporation (1 lab, W1). Vendors are referenced here not to imply differences in performance, but to provide a practical framework for capturing variability arising from differences in source design, ion optics, and analyzers that are inherently coupled to commercial platforms. In practice, the translation of a standardized method includes several other factors that may drive variation in ion species formation both within and between experiments. These data were used to evaluate the variability of ion species profiles across the 33 IRTS compounds using both negative and positive electrospray ionization. Of these compounds, 32 were detected in positive ion mode and 26 in negative ion mode.

### Variation of Ion Species across ESI-MS Vendor

We first evaluated the ion species profiles of the IRTS compounds by identifying the most abundant ion species per injection for each compound within each lab and aggregating the top ion species counts to determine the most abundant ion species for each instrument vendor. The choice to group by instrument vendor allowed for an account of the inherent differences in source design, ion optics, and analyzers that are coupled to commercial platforms. In practice, variation in ion species formation is also influenced by additional factors and does not correlate to any conclusions about the performance of individual instruments. These profiles were compared across each instrument vendor for data collected in both negative and positive ESI mode.

In negative mode, 19 of 26 (73%) IRTS compounds were detected as the same ion species across all vendors (5 [M + HCOO]^−^, 14 [M – H]^−^) (Figure 1B). The preferred ion species of the remaining seven IRTS compounds varied across instrument platforms. Of these, three exhibited a single outlier. For example, 259_XZAC was detected as [M – H]^−^ on all vendor instruments except Shimadzu, where this compound was not detected. This observation may be influenced by the ESI/APCI dual source design; however, definitive attribution is not possible, as other factors affecting ionization also vary across these comparisons.^18^ Similarly, 326_MADN and 484_FKDB were detected as [M – H]^−^ on all instruments except ThermoFisher and Shimadzu; respectively, where [M + HCOO]^−^ was favored by each. The IRTS compounds 345_VKUB and 393_SEKI exhibited a more variable pattern of ion species formation across instrument vendors, whereas neither was detected on the Shimadzu instruments, and [M + Cl]^−^ and [M + HCOO]^−^ were favored on the Thermo instruments. Additionally, for 345_VKUB, the most abundant ion form detected on Agilent instrumentation was [M + HCOO]^−^, making this the only IRTS compound to be observed as a different (i.e., not [M – H]^−^) most abundant ion species across all instrument vendors. Notably, for the three laboratories that reported solvent suppliers on Agilent instruments, the source and catalog number for LC–MS grade acetonitrile and methanol were identical, providing a potential explanation for this consistency outside of the instrumentation itself. Of the 19 IRTS compounds for which the same ion species was observed as most abundant across laboratories, at least two ion species were detected for each compound. Markedly, none of the calculated physicochemical properties (including LogP, polar surface area, and hydrogen acceptor/donor count) of the IRTS compounds were able to explain the patterns in ion species formation (Table S-1).^34^ While the variation of IRTS ion species is reported across vendors, it is also noteworthy that variation in ion species formation can arise from differences in method execution, solvent sources, and sample preparation. Across vendors, no two laboratories executed the standardized method in exactly the same way, emphasizing that the observed variability reflects the combined influence of instrumentation, solvent source, and laboratory practices.

Next, we considered the ion species profiles across instrument vendors using positive ESI. Here, 17 of the 32 (53%) IRTS compounds were detected as the same ion species ([M + H]^+^) across all instrumentation (Figure 1C). For the remaining 15 IRTS compounds, the most abundant ion species were variable across instrument platforms (among other varying factors). Notably, while this variability was observed for more compounds in positive ESI than negative ESI, the trends were similar. Namely, for eight of the IRTS compounds (230_MWSU, 239_QQKI, 339_YZKY, 393_SEKI, 400_OOAY, 484_FKDB, 553_YWXP, and 582_ULGJ), unique ion species formation was observed on one of the four commercial vendors. This uniqueness was most commonly observed on Waters instrumentation, where five of the aforementioned seven IRTS compounds were detected as a unique ion species compared to the other vendors. This ESI-MS vendor was only present in one lab and included variations external to instrumentation, including solvent supplier and sample preparation that may all also be related to this observation. The compound 339_YZKY was detected as [M + Na]^+^ on all instruments except ThermoFisher, where it was detected as [M + NH_4_]^+^, 582_ULGJ was detected as [M + Na]^+^ on all instruments except Agilent, where it was detected as [M + NH_4_]^+^, and 230_MWSU was detected as [M + Na]^+^ on all instruments except Shimadzu, where it was not detected. This missingness of specific IRTS compounds was again only observed on Shimadzu instruments and could reflect unique source design effects that reduce ionization efficiency for specific analytes.^18,49^ However, other effects driving these observations, including overall instrument sensitivity, contaminants, ion suppression, or in-source fragmentation, cannot be ruled out.^50,51^ Similarly, the variation in method execution across laboratories may also contribute to this observation.

Five of the IRTS compounds (259_MAYK, 304_PUZK, 306_OOPX, 314_XVJI, and 345_VKUB) showed dynamic ion species formation across at least 2 of the 4 instrument vendors. Specifically, 459_OFAM was detected as [M + Na]^+^ on Agilent and Waters, [M + H]^+^ on ThermoFisher, and [M + NH_4_]^+^ on Shimadzu. Similarly, 565_NYJF was detected as [M + Na]^+^ on Agilent and Waters, [M + NH_4_]^+^ on ThermoFisher, and was not detected on Shimadzu instruments. The dynamic preferences across both negative and positive mode ESI collectively indicate a complex and variable process of ion species formation for small molecules, that likely reflect the combined influences of instrumentation design and operation, as well as other sources of variability such as differences in method execution, solvent composition, sample preparation, instrument sensitivity, and potential in-source processes including fragmentation or ion suppression.

### Variation of Ion Species within ESI-MS Vendor

Unique ion species formation across different vendor instruments is not unexpected; however, we would anticipate less appreciable differences within vendor instruments. To explore this, we aggregated data across multiple laboratories utilizing instrumentation from the same vendor, allowing us to assess within-platform variability in instrument operation and method adoption. This included Shimadzu (2 laboratories: S1 and S2), ThermoFisher (3 laboratories: T1, T2 and T3), and Agilent (4 laboratories: A1, A2, A3, and A4). Results from Waters instrumentation were not included, as there was only one participating lab using a Waters instrument (W1).

We first assessed within-vendor consistency in the most abundant ion species formation (Figure 2A), and found that, in contrast to the diversity of preferred ion species observed across vendors, variability within a vendor was reduced. Namely, across the two Shimadzu laboratories (S1 and S2), the most abundant ion form was the same for all IRTS compounds. This was despite differences in IRTS preparation conditions and instrument models which may entail variations in sources, ion paths, and other components (Supporting Information Tables S-3, S-4 and S-7). Conversely, ion forms varied from ThermoFisher and Agilent instrumentation, with the 3 instruments in the ThermoFisher vendor group showing different preferred ion forms for five IRTS compounds. Similarly, the four laboratories using Agilent instrumentation also detected different preferred ion forms for five IRTS compounds. Two IRTS compounds, 326_MADN and 345_VKUB, had consistent ion forms within ThermoFisher and Agilent systems (Figure 2B). The remaining IRTS compounds that had variable ion profiles were unique to ThermoFisher (304_PUZK, 393_SEKI, and 454_FKDB) or Agilent (314_XVJI, 400_OOAY, 459_OFAM) instrumentation.

**Figure 2:**
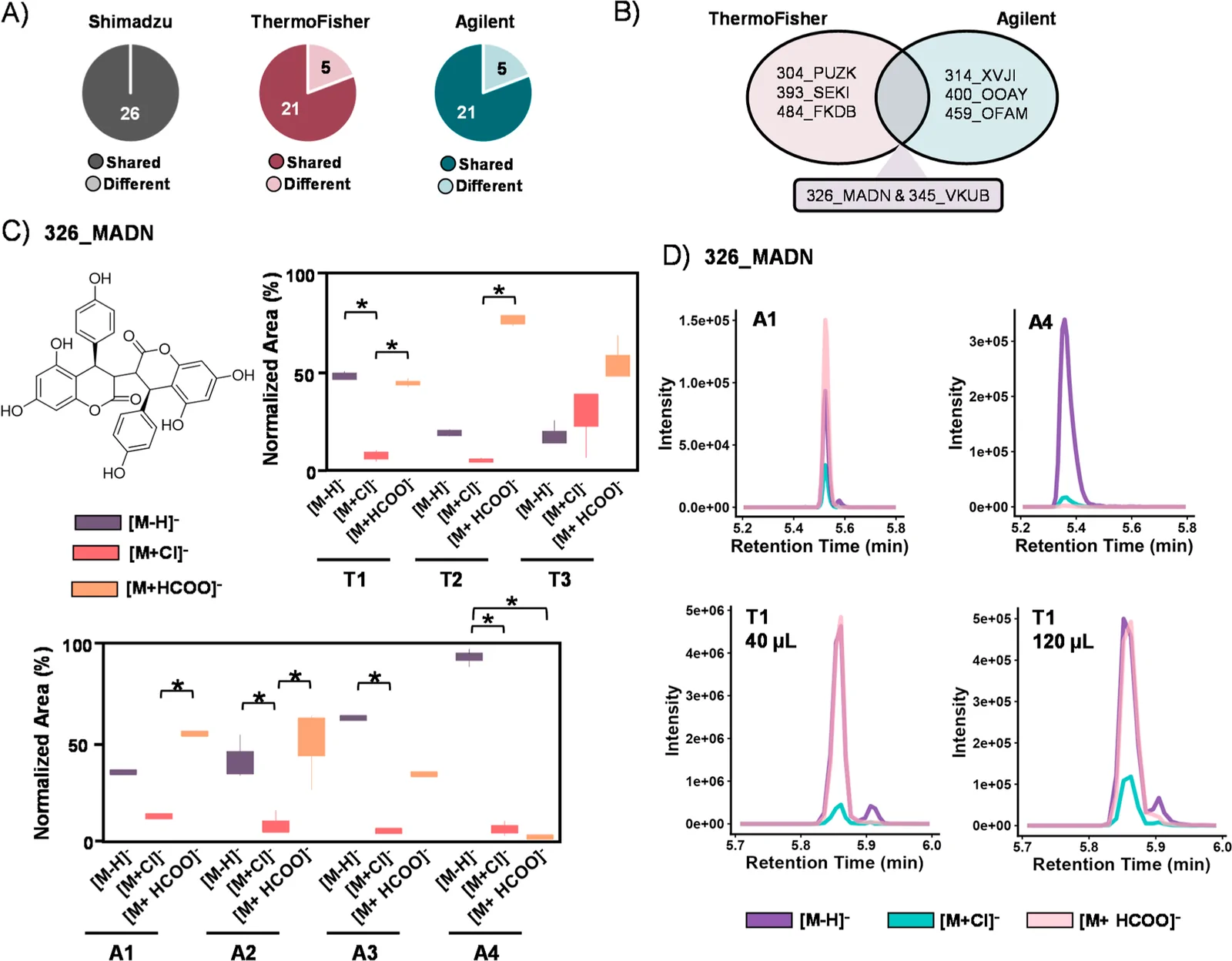
Ion species formation within commercial instrument vendors in negative mode ESI. (A) Pie charts reflecting the profile of adduct species formation for all IRTS compounds within Shimadzu (2 laboratories, left), ThermoFisher (3 laboratories, middle), and Agilent (4 laboratories, right) instruments. The darker color of the pie indicates the proportion of IRTS compounds for which the same ion species was observed across laboratories. The lighter color of the pie reflects the proportion of IRTS compounds for which the ion species was different across laboratories. (B) A Venn diagram of the specific IRTS compounds for which the ion species were different across laboratories: Thermo instrumentation (left), Agilent instrumentation (right). (C) Normalized box plots of the ion species abundance (plotted as area) for the IRTS compound 326_MADN within the three ThermoFisher laboratories (left) and the four Agilent laboratories (right). Significant differences (*p*-value ≤0.05) in ion species within each instrument vendor is denoted with an asterisk.^46–48^ (D) Extraction ion chromatograms (EICs) for the ion species of 326_MADN from laboratories A1 and A4. EIC for the ion species of 326_MADN in lab T1: high concentration (left), low concentration (right).

From this, we next sought to understand how the ion species profiles varied across laboratories using instruments from the same vendor, though the models, operation settings, and other method adoption variables still varied. We analyzed data from both ThermoFisher and Agilent laboratories using the ion species profile of the IRTS compound 326_MADN. Within each injection, ion areas were normalized and plotted to depict the variation of ion species abundance within each lab. Across the laboratories using ThermoFisher instrumentation, the data show several unique features in 326_MADN negative ESI ion species formation (Figure 2C). Specifically, in lab T1, [M – H]^−^ and [M + HCOO]^−^ were both highly abundant, averaging 48.1 and 44.4% of the total 326_MADN signal, respectively. This was significantly more abundant than [M + Cl]^−^ which represented 7.5% of the total 326_MADN signal. Conversely, in lab T2, [M + HCOO]^−^ represented 76.5% of the total 326_MADN signal, while the [M – H]^−^ and [M + Cl]^−^ ion species were significantly lower, at 18.8% and 4.8%; respectively. In lab T3, [M + HCOO]^−^ was again the most abundant ion species, accounting for 54.8% of 326_MADN total ion signal. There was no significant difference in abundance between this ion species and the [M – H]^−^ (17.6%) and [M + Cl]^−^ (27.6%) ion species. Normalized ion species profiles of 326_MADN also varied across laboratories using Agilent instrumentation. Specifically, in lab A1, [M + HCOO]^−^ accounted for 54.2% of the total 326_MADN signal. This was significantly more than [M + Cl]^−^ at 11.5% but not significantly more than the [M – H]^−^ ion species, which made up the remaining 34.3%. Lab A2 showed a similar ion species distribution of 326_MADN, with relative intensities of the total 326_MADN signal, averaging 51.7%, 41.1% and 7.2% for [M + HCOO]^−^, [M – H]^−^, and [M + Cl]^−^; respectively. Conversely, instrumentation in laboratories A3 and A4 showed preference for the [M – H]^−^ ion species. For lab A3, [M – H]^−^ accounted for 62.6% of the total 326_MADN signal. This was significantly more than [M + Cl]^−^ at 3.9%, but not significantly more than the [M – H]^−^ ion species, which made up the remaining 33.5%. In lab A4, [M – H]^−^ was the most abundant, averaging 93.5% of the total 326_MADN signal. This was significantly more than both [M + Cl]^−^ and [M – H]^−^, which were 6.1% and 0.6%; respectively. Across all Agilent and ThermoFisher instrumentation, differences in instrument models and operating parameters (e.g., voltages, temperatures, solvent supplier source) could contribute to the observed variation in ion species formation.^22,30^ Notably, lab A4 was the only system to implement an Agilent 6560, which includes an additional drift tube ion mobility stage that may contribute to the notable absence of the [M + HCOO]^−^ adduct species.^52–54^

To contextualize these patterns, we examined representative ion species from Agilent and ThermoFisher instrumentation to highlight both shared and vendor-specific features. Lab A1 displayed a profile dominated by [M + HCOO]^−^, with [M – H]^−^ and [M + Cl]^−^ present at much lower abundance. In contrast, lab A4 exhibited a pronounced inversion, where [M – H]^−^ was the most abundant ion species (Figure 2D). Dilution series data from Lab T1 offered additional insight into a concentration-dependent shift in ion species intensity. Namely, at lower analyte concentrations, the formate ion species predominated, whereas at higher concentrations the deprotonated species became dominant. These results demonstrate that, beyond interlab differences, the most abundant ion species can also be influenced by analyte concentration. Other work has demonstrated similar concentration-dependent ion formation preferences for piperidine analogues, adding another layer of complexity for translating the factors driving ion species formation in nontargeted and semitargeted small molecule applications.^55^

This analysis was also conducted for ion species formation in positive mode ESI. We observed variations in ion species profiles across all three vendors (Figure 3A). For Shimadzu instruments (two laboratories: S1 and S2), the most abundant ion species varied for two IRTS compounds, while for both ThermoFisher instruments (three laboratories: T1, T2 and T3) and Agilent instruments (four laboratories: A1, A2, A3, and A4), the most abundant ion species varied for 11 IRTS compounds. No IRTS compounds showed variable ion species profiles for all three vendor instruments (Figure 3B). However, six IRTS compounds showed variation in ion species formation across Agilent and Thermo laboratories. These included 256_MAYK, 314_XVJI, 345_VKUB, 459_OFAM, 484_FKDB, and 582_ULGJ. Shimadzu laboratories showed unique variation in ion species formation for only one IRTS compound, 326_MADN. Notably, the ion species formation of this compound was variable across Agilent and ThermoFisher instruments in negative mode as well. ThermoFisher laboratories showed variability in the most abundant ion species for four IRTS compounds: 230_MWSU, 304_PUZK, 332_YZKY, and 565_NYJF. Similarly, the four Agilent laboratories showed variability in the most abundant ion species for five IRTS compounds: 201_BWJD, 239_QQKI, 393_SEKI, 400_OOAY, and 553_YWXP. Notably, the preferred ion species observed for 304_PUZK and 400_OOAY was also variable in negative mode. Overall, these results reveal greater variation in ion species formation in positive ESI compared to negative ESI.^30^

**Figure 3:**
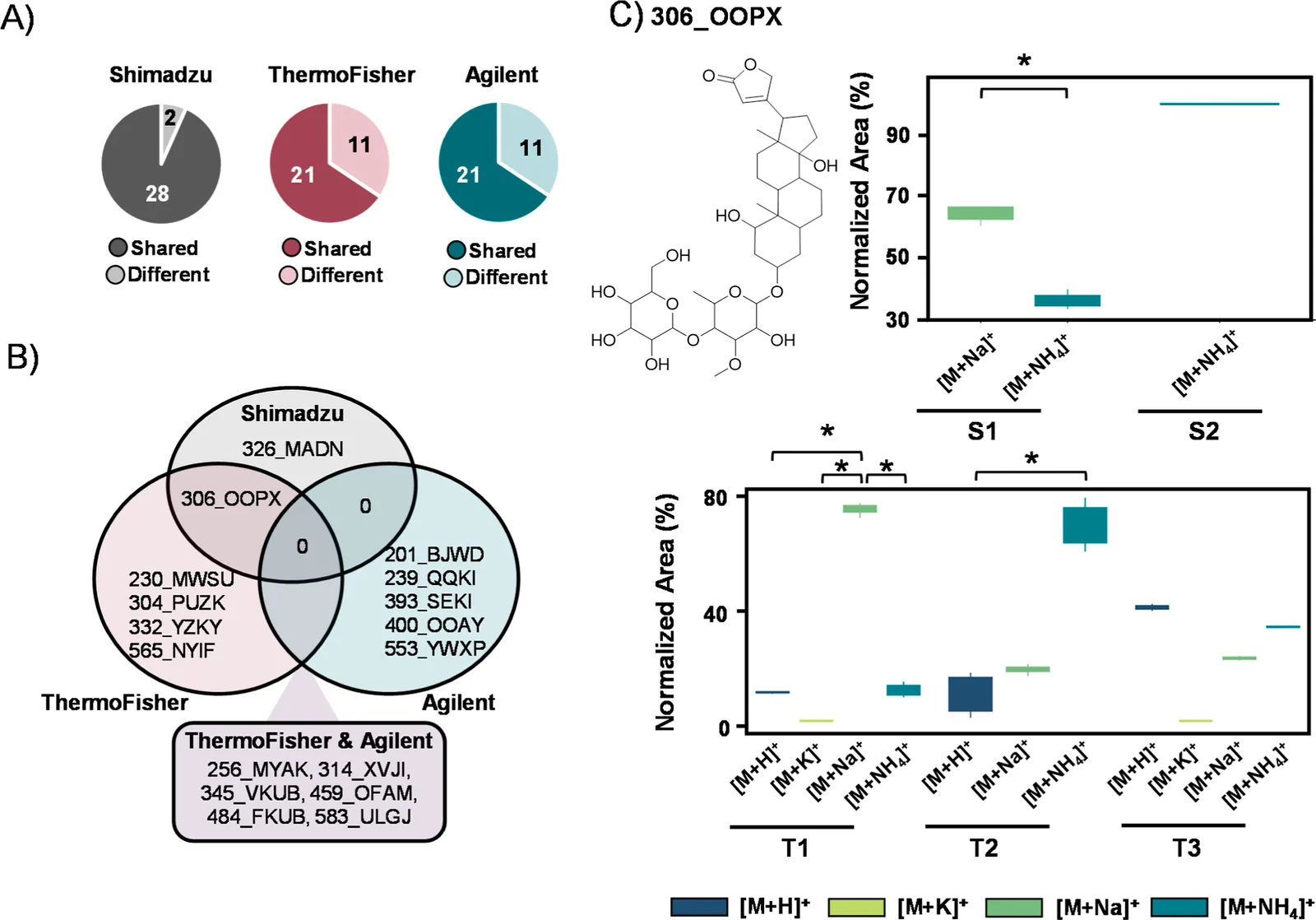
Ion species formation within commercial instrument vendors in positive mode ESI. (A) Pie charts reflecting the profile of ion species formation for all IRTS compounds within Shimadzu (2 laboratories, left), ThermoFisher (3 laboratories, middle), and Agilent (4 laboratories, right) instruments. The darker color of the pie indicates the proportion of IRTS compounds for which the same ion species was observed across laboratories. The lighter color of the pie reflects the proportion of IRTS compounds for which the ion species was different across laboratories. (B) A Venn diagram of the specific IRTS compounds for which the ion species were different across laboratories: ThermoFisher instrumentation (left), Agilent instrumentation (right), and Shimadzu instrumentation (top). (C) Normalized box plots of the ion species abundance for the IRTS compound 306_OOPX across two Shimadzu laboratories (top) and three ThermoFisher laboratories (bottom). Significant differences (*p*-value ≤0.05) in ion species within each instrument vendor is denoted with an asterisk.^46–48^

Focusing on the IRTS compound 306_OOPX, we further explored the variation of ion species formation within Shimadzu and ThermoFisher instrumentation. In data from the two Shimadzu instruments, we detected unique ion species (Figure 3C), where in S1 [M + Na]^+^ was most abundant, averaging 69.6% of the 306_OOPX total signal and [M + NH_4_]^+^ accounting for the remaining 36.4%. In S2, the only observed ion species for 306_OOPX was [M + NH_4_]^+^. While Shimadzu instruments were generally characterized by the relative simplicity in their ion species profiles for 306_OOPX, it should be noted that this work does not consider any ion clusters, multimers, or in-source fragmentation that could also account for the variability in ion channels observed for 306_OOPX.^55,56^ In the three ThermoFisher laboratories, ion species profiles were again unique to each lab (Figure 3D). Specifically, lab T1 observed four ion species: [M + H]^+^, [M + K]^+^, [M + Na]^+^, and [M + NH_4_]^+^, with [M + Na]^+^ being significantly more abundant (75.1%) than all other ion species. In lab T2, all ion species except [M + K]^+^ were detected with [M + NH_4_]^+^ being the most abundant, accounting for 69.1% of the total 306_OOPX signal. This was significantly more than [M + H]^+^ (11.6%), but not [M + Na]^+^ (19.4%). Finally, lab T3 detected all four ion species with no significant differences, likely due to a low number of replicates for this data set. The total signal distributions were: [M + H]^+^ (41.0%) > [M + NH_4_]^+^ (34.3%) > [M + Na]^+^ (23.3%) > [M + K]^+^ (1.4%). While the positive ion mode data reflects a similar uniqueness of the most abundant ion species of individual IRTS compounds compared to negative ion mode, the trends in positive ion mode were exaggerated, with not only significant differences in ion species profiles across laboratories, but also the varied presence/absence of ion species across instruments. This lack of consistency in ion species formation across instrumentation further complicates the use of a single “consensus” ion species for identifying and reporting analytes in small molecule applications, highlighting the use of ion species grouping with methods such as RAMClust, MZMine, and CAMERA.^57–59^

Fluctuations in ion species formation across negative and positive ESI present a meaningful challenge for standardized reporting in small molecule applications. The results presented here indicate that the collective influence of controllable factors such as source or MS settings, and uncontrollable factors such as commercial instrument design and general, subtle differences in solvent composition or sample handling can make ion species formation inconsistent, even across the same vendor (both within and between laboratories). In these data, the prevalence of a specific ion species ranged over 65% instrument-to-instrument, even within the same vendor. These observations suggest that small variations, often introduced without concern when implementing a given method, may have a disproportionately large impact on ion species formation. While evaluation of the IRTS compound mix provides a lens into these variations, it is important to note that in real-world small molecule applications where the preferred ion species form is often uncharacterized, these differences could obscure accurate annotation and quantification.

### Variation of Ion Species Formation within Laboratories

To distinguish instrument-driven effects from interlaboratory differences, we considered ion species variation within individual laboratories through evaluation of multiple injections on the same instrument, effectively reducing variation from differences in standardized method adoption across laboratories. This analysis revealed fluctuations in the most abundant negative mode ion species of one or more IRTS compounds in 6 out of 10 laboratories (Figure 4A). In positive mode, 9 of 10 laboratories saw a change in the most abundant ion species. Within the instrument vendors, we also observed varied formation of these ion species. Namely, Shimadzu and ThermoFisher instruments showed the greatest agreement of the most abundant ion species, whereas Waters and Agilent instruments showed the most disagreement among or between the most abundant ion species. To understand this further, we considered how the data was collected within each lab. In 7 of 10 laboratories, replicate data was collected across separate acquisition periods (e.g., nonconsecutive days spanning several weeks or months between acquisition periods) and therefore may have used different mobile phase preparations or instrument performance, which may contribute to inconsistencies in preferred ion form within a lab. For the remaining three laboratories, W1, A1, and A4, data was collected in consecutive acquisitions (e.g., same or consecutive days where systems were cleaned, mobile phases added, etc. akin to routine, large experiment studies). For these cases, variation of ion species formation was still observed in positive mode for several IRTS compounds. Lab A4 also observed variation in ion species formation in negative mode.

**Figure 4:**
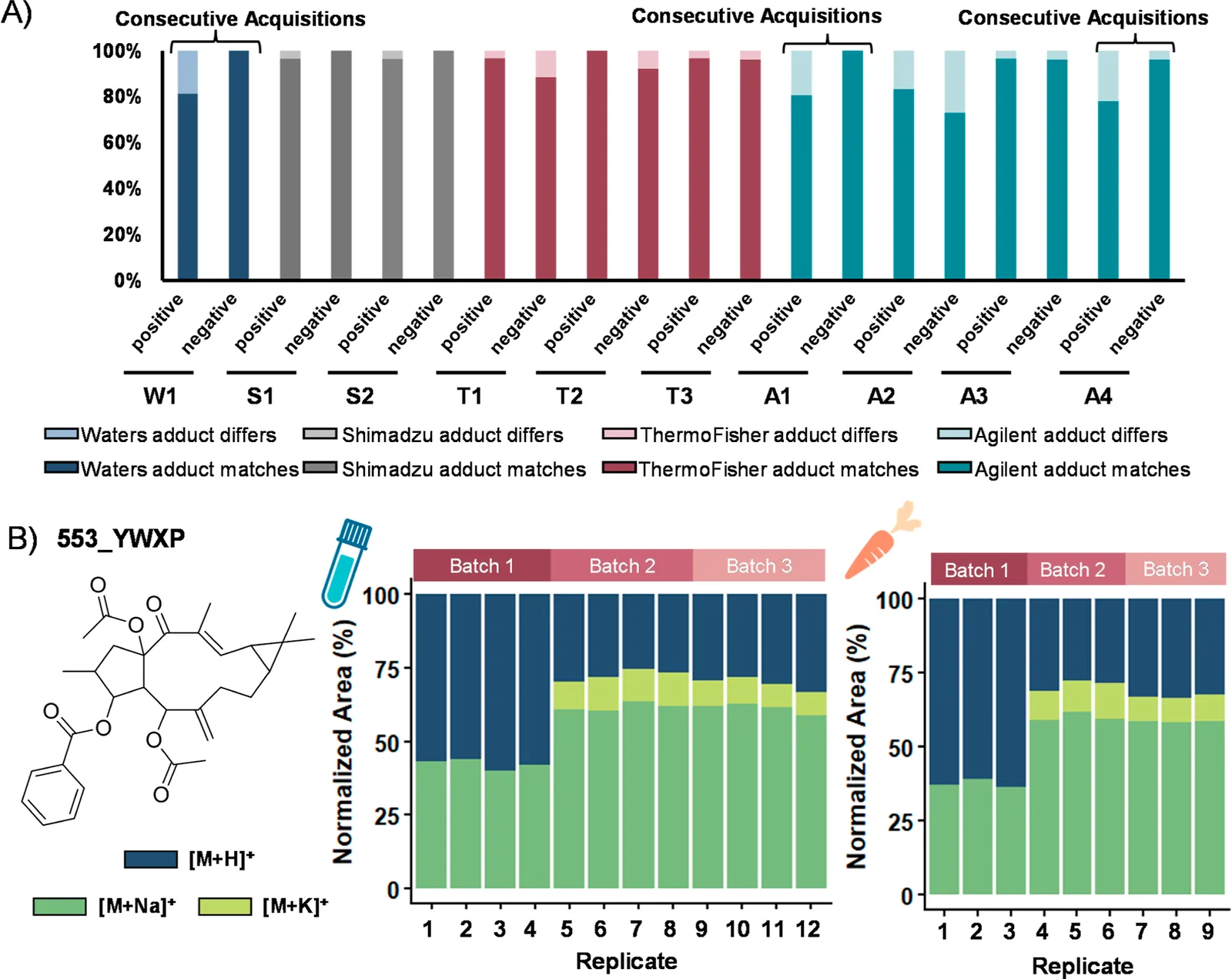
Within-laboratory consensus of ion species. (A) Stacked bar chart of the most abundant ion species in positive and negative ESI for each laboratory. Results are colored by the instrument vendor (Waters, blue; Shimadzu, gray; Thermo, red; and Agilent, teal). (B) A stacked bar chart of the normalized % area of observed ion species for 553_YWXP in each IRTS blank injection completed by lab A1 for blank (left) and carrot (right). The colors correspond to the ion species ([M + H]^+^, blue; [M + Na]^+^, green; and [M + K]^+^, lime green).

We next sought to define the quantitative impact of these variations by comparing the ion species formation profiles of a single IRTS compound across replicate IRTS blank injections within one lab (Figure 4B). Two distinct profiles emerged: in four injections, [M + H]^+^ was the most abundant ion species (58.3–56.1%), followed by [M + Na]^+^ (43.8–40.6%). In the remaining eight injections, three ion species were observed, with [M + Na]^+^ as the most abundant (62.7–60.1%), followed by [M + H]^+^ (31.2–26.4%) and [M + K]^+^ (11.0–8.2%). Similar trends were observed for this compound in a complex matrix background of orange carrot, where interferences from coeluting analytes can influence ion generation pathways.^60^ In three injection replicates, [M + H]^+^ was the most abundant (63.7–61.1%), followed by [M + Na]^+^ (38.8–36.2%), and in the remaining six injection replicates [M + Na]^+^ was the most abundant (60.4–58.0%), followed by [M + H]^+^ (33.4–28.5%), and [M + K]^+^ (11.1–8.3%) (Figure 4C). Notably, the two patterns of ion species profiles also corresponded to the acquisition timeline, with the earlier analyses including two ion species and later injections observing three. This analysis, which was a part of a larger (>100 samples) study that was analyzed through three experimental batches, showed a clear difference in batch 1 compared to batches 2 and 3 that correspond with ion profile differences. Between each acquisition batch, the source was cleaned and mobile phases were refreshed. After batch 2, the analytical column was also replaced. While these procedural changes temporally align with the observed differences, a direct causal relationship cannot be established given the number of variations. Additionally, this observation is consistent with other reports that show the stabilization time of MS spectra for specific analytes can take several hours.^61^ Nevertheless, these variations are noteworthy for both annotation and quantification, as they illustrate cases where relying on a single ion species may misrepresent the data, given that the most abundant ion species can vary between injections.^62,63^

With influence from external variation of solvent supplier and method adoption controlled within laboratories, we sought to next consider if artifact signals could possibly be conflating ion species observations within a lab. To further understand these variations, we examined ion mobility data collected on an Agilent 6560 IM-qTOF by laboratory A4 (Figure 5A). Across the seven IRTS compounds with observed variation in ion species formation (one detected in negative ESI, six detected in positive ESI), five compounds exhibited the presence of two or more unique drift times for one ion species. The observation of multiple peaks in the ion mobility data may suggest the presence of an interfering signal that is contributing to the intensity of this ion species. However, these samples contained only solvent and the IRTS compounds, which would suggest any isobaric interferences result from a contamination or artifact signal. A more likely explanation is the presence of unique 3D conformations of the IRTS compounds (conformers) or protonation at different sites (protomers), creating charge isomers for a single IRTS compound. For the other two IRTS compounds, 400_OOAY and 459_OFAM, only one drift time for each ion species was observed, suggesting that either isomeric ion species are not present, or not resolved with the relatively low resolving power of the DTIMS separation (<60), which may obscure the presence of ion configurations with small structural differences.^42^

**Figure 5:**
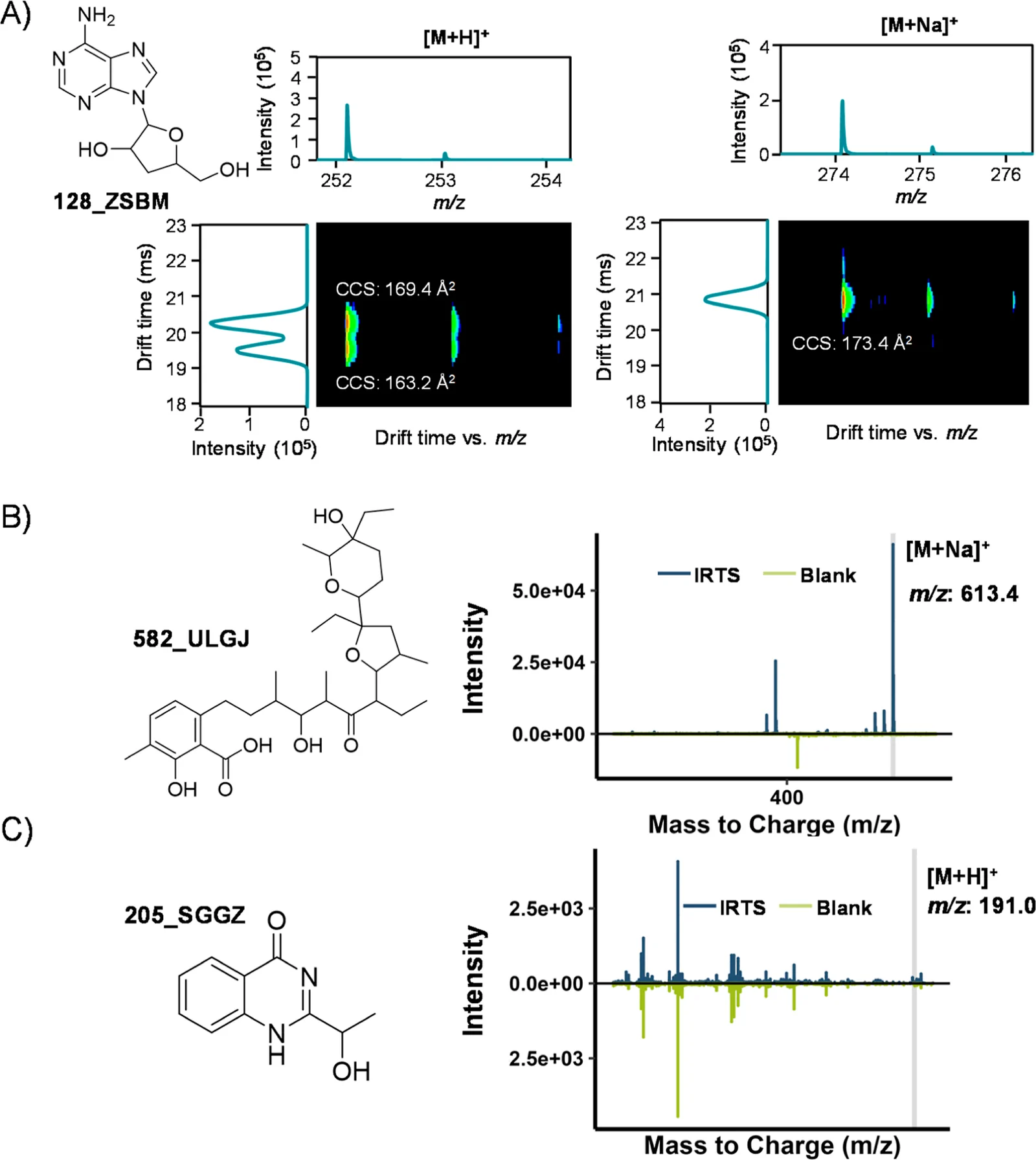
(A) IMS profile data of 128_ZSBM protonated and sodiated ion species; spectrum (top), drift time distributions of the mass range (left), heatmap of the ion mobility data within the mass and drift time spaces (bottom). (B) 582_ULGJ MS/MS spectra in IRTS sample (top) and 80:20 MeOH H_2_O (inverted spectra) at retention time 9.1 min. The gray highlighted regions represent the isolated ion species (sodiated, [M + Na]^+^). (C) 205_SGGZ MS/MS spectra in IRTS sample (top) and 80:20 methanol/H_2_O (inverted spectra) at retention time 3.1 min. The gray highlighted regions represent the [M + H]^+^ ion species. Additional IRTS are in Supporting Information Figures S1–S12.

For IRTS compounds exhibiting variable ion species formation on the W1 instrument, confirmatory analysis using LC–MS/MS (data dependent acquisition) was performed using inclusion lists for each ion species. Fragmentation of the *m*/*z* corresponding to the [M + Na]^+^ ion species of 582_ULGJ resulted in a distinct fragmentation pattern in the IRTS sample compared to a solvent blank (Figure 5B). This behavior was consistent for all evaluated ion species for the IRTS compounds with one exception. Similar fragmentation patterns were observed in both the IRTS sample and the solvent blank when isolating the *m*/*z* corresponding to the [M + H]^+^ ion species of 205_SGGZ (Figure 5C). Although this could be a consequence of sample carryover, the absence of similar behavior in other IRTS compounds and the restriction of this observation to a single ion species of 205_SGGZ suggests that carryover is unlikely. Instead, this observation suggests that the apparent [M + H]^+^ signal for 205_SGGZ in the IRTS sample may be confounded by background contributions from the solvent or other artifacts. Collectively, the confirmatory IMS and MS/MS data indicate the potential presence of coeluting, unidentified interferences that complicate the accurate summation of ion species intensities, posing challenges for quantitative comparisons through a summation of all observed ions comprehensive analyte identification.

## Conclusions

The influence of controllable and uncontrollable factors on ion species formation holds importance for the application of LC–ESI–MS to scientific challenges. For nontargeted applications with unknown “true” answers, the fundamental variations driving ion species preference presents obstacles for both identification and quantification of small molecules. These challenges extend to semitargeted approaches where stable isotope labeled internal standards are not available for every compound. Here, we provide critical insights into the effect of ion species generation across 10 laboratories with instrumentation from Agilent, Shimadzu, ThermoFisher, and Waters, which analyzed identical samples using standardized methods adopted with slight variations within each lab. Across this network, the most abundant ion species observed in both negative and positive ion mode was found to vary across instruments, an unsurprising outcome given the differences in ion source configuration and operating conditions across platforms coupled to the variations in how laboratories adopted the method.

Continued exploration, however, revealed that differences in preferred ion species also occurred between laboratories using instruments from the same vendor. Even more surprisingly, these differences were present within data from a single laboratory and even within consecutive experimental acquisitions common for large experiments. Confirmatory IMS and DDA analysis revealed potential background interferents that could account for or contribute to these observations for some, but not all, observations, indicating a complex interplay between instrumentation, ionization environment, and preferential ion species formation pathways. Some molecular features exhibited recurring patterns of ion species variation across acquisitions and laboratories, while others appeared only once under a single set of conditions. For molecules without structural identification, the recurring and transient ion species variations reflect a combination of stochastic and context-specific processes driving small molecule ionization. These findings, while essential for analyte identification, also have significant implications for qualitative and quantitative reporting both within and across laboratories. As data repositories and method standardization efforts grow in omics, the data presented here underscore the challenges in relying on a single “high confidence” ion species or summing ion species for reporting reliable, comparable data. While method standardization may improve consistency, our results indicate that fully harmonized ionization behavior across real-world laboratory conditions remains challenging due to the influence of controlled and uncontrolled factors, as well as artifactual contributions that skew trends. As such, database efforts should prioritize the incorporation of data from multiple platforms and experimental conditions to better capture the diversity of ion species that may be observed. In this context, comprehensive metadata reporting, feature grouping across adducts, and inclusion of multiple ion species per compound will be critical for improving database robustness and enabling more reliable cross-study comparisons. Concurrently, systematically evaluating, recognizing and addressing ion species variability in ESI–MS applications to elucidate the contributions of individual variables and ESI alignment is essential. Taken together, these findings emphasize that embracing, rather than oversimplifying, ion species complexity is essential for unlocking reproducible, comparable LC–ESI-MS data sets across platforms and laboratories.

## Supplementary Material





## Notes

Instrumentation properties, data outputs, and statistical analyses completed in this work are available through the corresponding Supporting Information. R code for data analysis is available at Github at: https://github.com/ThePrenniLab/Odenkirk_Adducts_Across_Vendors. Raw spectra and extracted ion chromatograms (EICs) of data are available at: https://panoramaweb.org/5co3dQ.url (PDF)
Odenkirk_JASMS_MulitiLab_Ion_Supporting Information_revised_260304 (XLSX)

## Abbreviations

ESI: electrospray ionization
MS: mass spectrometry
IRTS: internal retention time standard
EIC: extracted ion chromatogram
CCS: collision cross section
DDA: data dependent acquisition
IMS: ion mobility spectrometry

## References

[1] Yamashita M., Fenn J. B. (1984). Electrospray Ion Source. Another Variation on the Free-Jet Theme. J. Phys. Chem..

[2] Banerjee S., Mazumdar S. (2012). Electrospray Ionization Mass Spectrometry: A Technique to Access the Information beyond the Molecular Weight of the Analyte. Int. J. Anal. Chem..

[3] Khalikova M., Jireš J., Horáček O., Douša M., Kučera R., Nováková L. (2024). What Is the Role of Current Mass Spectrometry in Pharmaceutical Analysis?. Mass Spectrom. Rev..

[4] Liu Z., Zhang M., Chen P., Harnly J. M., Sun J. (2022). Mass Spectrometry-Based Nontargeted and Targeted Analytical Approaches in Fingerprinting and Metabolomics of Food and Agricultural Research. J. Agric. Food Chem..

[5] Griffiths W. J., Wang Y. (2009). Mass Spectrometry: From Proteomics to Metabolomics and Lipidomics. Chem. Soc. Rev..

[6] Deng Y.-T., You J., He Y., Zhang Y., Li H.-Y., Wu X.-R., Cheng J.-Y., Guo Y., Long Z.-W., Chen Y.-L., Li Z.-Y., Yang L., Zhang Y.-R., Chen S.-D., Ge Y.-J., Huang Y.-Y., Shi L.-M., Dong Q., Mao Y., Feng J.-F., Cheng W., Yu J.-T. (2025). Atlas of the Plasma Proteome in Health and Disease in 53,026 Adults. Cell.

[7] Leutert M., Entwisle S. W., Villén J. (2021). Decoding Post-Translational Modification Crosstalk With Proteomics. Mol. Cell. Proteomics.

[8] Vernocchi P., Del Chierico F., Putignani L. (2016). Gut Microbiota Profiling: Metabolomics Based Approach to Unravel Compounds Affecting Human Health. Front. Microbiol..

[9] Guasch-Ferré M., Bhupathiraju S. N., Hu F. B. (2018). Use of Metabolomics in Improving Assessment of Dietary Intake. Clin. Chem..

[10] Lydic T. A., Goo Y. (2018). Lipidomics Unveils the Complexity of the Lipidome in Metabolic Diseases. Clin. Transl. Med..

[11] Allwood J. W., Williams A., Uthe H., Van Dam N. M., Mur L. A. J., Grant M. R., Pétriacq P. (2021). Unravelling Plant Responses to Stress—The Importance of Targeted and Untargeted Metabolomics. Metabolites.

[12] Dominguez-Medina S., Fostner S., Defoort M., Sansa M., Stark A.-K., Halim M. A., Vernhes E., Gely M., Jourdan G., Alava T., Boulanger P., Masselon C., Hentz S. (2018). Neutral Mass Spectrometry of Virus Capsids above 100 Megadaltons with Nanomechanical Resonators. Science.

[13] Wolff, P. ; Ennifar, E. Native Electrospray Ionization Mass Spectrometry of RNA-Ligand Complexes. In RNA Spectroscopy; Arluison, V. , Wien, F. , Eds.; Springer US: New York, NY, 2020; Vol. 2113, pp 111–118. 10.1007/978-1-0716-0278-2_9 32006311

[14] Leney A. C., Heck A. J. R. (2017). Native Mass Spectrometry: What Is in the Name?. J. Am. Soc. Mass Spectrom..

[15] Meher A. K., Chen Y.-C. (2017). Electrospray Modifications for Advancing Mass Spectrometric Analysis. Mass Spectrom..

[16] Mordehai, A. ; Fjeldsted, J. Agilent Jet Stream Thermal Gradient Focusing Technology; Santa Clara, 2009 .

[17] User Guide: HESI-II Probe.

[18] Brecht D., Uteschil F., Schmitz O. J. (2020). Development of a Fast-switching Dual (ESI/APCI) Ionization Source for Liquid Chromatography/Mass Spectrometry. Rapid Commun. Mass Spectrom..

[19] Manisali I., Chen D. D. Y., Schneider B. B. (2006). Electrospray Ionization Source Geometry for Mass Spectrometry: Past, Present, and Future. TrAC, Trends Anal. Chem..

[20] Kiontke A., Oliveira-Birkmeier A., Opitz A., Birkemeyer C. (2016). Electrospray Ionization Efficiency Is Dependent on Different Molecular Descriptors with Respect to Solvent PH and Instrumental Configuration. PLoS One.

[21] Kruve A. (2016). Influence of Mobile Phase, Source Parameters and Source Type on Electrospray Ionization Efficiency in Negative Ion Mode. J. Mass Spectrom..

[22] Page J. S., Kelly R. T., Tang K., Smith R. D. (2007). Ionization and Transmission Efficiency in an Electrospray Ionization—Mass Spectrometry Interface. J. Am. Soc. Mass Spectrom..

[23] Heintz C., Kersten H., Benter T., Wißdorf W. (2025). Signatures of Charged Droplets from ESI: A Statistical Analysis of Non-Summed Mass Spectra Compared to APCI. J. Am. Soc. Mass Spectrom..

[24] Liigand J., Kruve A., Leito I., Girod M., Antoine R. (2014). Effect of Mobile Phase on Electrospray Ionization Efficiency. J. Am. Soc. Mass Spectrom..

[25] Mallet C. R., Lu Z., Mazzeo J. R. (2004). A Study of Ion Suppression Effects in Electrospray Ionization from Mobile Phase Additives and Solid-phase Extracts. Rapid Commun. Mass Spectrom..

[26] Liu B., Tang Z., Huan T. (2025). Adduct-Induced Variability in Tandem Mass Spectrometry. Anal. Chem..

[27] Calixte E. I., Liyanage O. T., Gass D. T., Gallagher E. S. (2021). Formation of Carbohydrate–Metal Adducts from Solvent Mixtures during Electrospray: A Molecular Dynamics and ESI-MS Study. J. Am. Soc. Mass Spectrom..

[28] Erngren I., Haglöf J., Engskog M. K. R., Nestor M., Hedeland M., Arvidsson T., Pettersson C. (2019). Adduct Formation in Electrospray Ionisation-Mass Spectrometry with Hydrophilic Interaction Liquid Chromatography Is Strongly Affected by the Inorganic Ion Concentration of the Samples. J. Chromatogr. A.

[29] Marwah P., Marwah A. K., Zimba P. V. (2020). Controlling Formation of Metal Ion Adducts and Enhancing Sensitivity in Liquid Chromatography Mass Spectrometry. J. Appl. Nat. Sci..

[30] Konermann L., Ahadi E., Rodriguez A. D., Vahidi S. (2013). Unraveling the Mechanism of Electrospray Ionization. Anal. Chem..

[31] Kruve A., Kaupmees K. (2017). Adduct Formation in ESI/MS by Mobile Phase Additives. J. Am. Soc. Mass Spectrom..

[32] Stricker T., Bonner R., Lisacek F., Hopfgartner G. (2021). Adduct Annotation in Liquid Chromatography/High-Resolution Mass Spectrometry to Enhance Compound Identification. Anal. Bioanal. Chem..

[33] Odenkirk M. T., Michel C., Doenges K. A., Leaptrot K. L., Sherrod S. D., May J. C., Read M. L., Chaparro J. M., Montgomery N., Brinkley S., Vaniya A., Mitchell S. B., Yu B., Elhaderi J. K., Henson L., Broeckling C. D., Fiehn O., Reisdorph R., Reisdorph N., McLean J. A., Chien C.-M., Shafizadeh T., De La Parra J., Ahmed S., Watkins S., Prenni J. E. (2025). A Standardized Nontargeted Metabolomics Method for Cross-Laboratory Comparison of Food Profiles. Food Chem..

[34] RDKit: Open-Source Cheminformatics.

[35] Standardized Omics at a Global Scale: A Perspective on Barriers, Opportunities, and Lessons Learned.

[36] May J. C., Knochenmuss R., Fjeldsted J. C., McLean J. A. (2020). Resolution of Isomeric Mixtures in Ion Mobility Using a Combined Demultiplexing and Peak Deconvolution Technique. Anal. Chem..

[37] Belov M. E., Clowers B. H., Prior D. C., Danielson Iii W. F., Liyu A. V., Petritis B. O., Smith R. D. (2008). Dynamically Multiplexed Ion Mobility Time-of-Flight Mass Spectrometry. Anal. Chem..

[38] Adams K. J., Pratt B., Bose N., Dubois L. G., St. John-Williams L., Perrott K. M., Ky K., Kapahi P., Sharma V., MacCoss M. J., Moseley M. A., Colton C. A., MacLean B. X., Schilling B., Thompson J. W. (2020). Skyline for Small Molecules: A Unifying Software Package for Quantitative Metabolomics. J. Proteome Res..

[39] Zhang Y., Liu F., Li X. Q., Gao Y., Li K. C., Zhang Q. H. (2024). Generic and Accurate Prediction of Retention Times in Liquid Chromatography by Post–Projection Calibration. Commun. Chem..

[40] Sharma V., Eckels J., Schilling B., Ludwig C., Jaffe J. D., MacCoss M. J., MacLean B. (2018). Panorama Public: A Public Repository for Quantitative Data Sets Processed in Skyline. Mol. Cell. Proteomics.

[41] Bilbao A., Gibbons B. C., Stow S. M., Kyle J. E., Bloodsworth K. J., Payne S. H., Smith R. D., Ibrahim Y. M., Baker E. S., Fjeldsted J. C. (2022). A Preprocessing Tool for Enhanced Ion Mobility–Mass Spectrometry-Based Omics Workflows. J. Proteome Res..

[42] Stow S. M., Causon T. J., Zheng X., Kurulugama R. T., Mairinger T., May J. C., Rennie E. E., Baker E. S., Smith R. D., McLean J. A., Hann S., Fjeldsted J. C. (2017). An Interlaboratory Evaluation of Drift Tube Ion Mobility–Mass Spectrometry Collision Cross Section Measurements. Anal. Chem..

[43] Kessner D., Chambers M., Burke R., Agus D., Mallick P. (2008). ProteoWizard: Open Source Software for Rapid Proteomics Tools Development. Bioinformatics.

[44] R: A Language and Environment for Statistical Computing.

[45] Kruskal W. H., Wallis W. A. (1952). Use of Ranks in One-Criterion Variance Analysis. J. Am. Stat. Assoc..

[46] Dunn O. J. (1964). Multiple Comparisons Using Rank Sums. Technometrics.

[47] Benjamini Y., Hochberg Y. (1995). Controlling the False Discovery Rate: A Practical and Powerful Approach to Multiple Testing. J. Roy. Stat. Soc. B Stat. Methodol..

[48] Oss M., Kruve A., Herodes K., Leito I. (2010). Electrospray Ionization Efficiency Scale of Organic Compounds. Anal. Chem..

[49] Demarque D. P., Crotti A. E. M., Vessecchi R., Lopes J. L. C., Lopes N. P. (2016). Fragmentation Reactions Using Electrospray Ionization Mass Spectrometry: An Important Tool for the Structural Elucidation and Characterization of Synthetic and Natural Products. Nat. Prod. Rep..

[50] Metz T. O., Baker E. S., Schymanski E. L., Renslow R. S., Thomas D. G., Causon T. J., Webb I. K., Hann S., Smith R. D., Teeguarden J. G. (2017). Integrating Ion Mobility Spectrometry Into Mass Spectrometry-Based Exposome Measurements: What Can It Add and How Far Can It Go?. Bioanalysis.

[51] te Brinke E., Arrizabalaga-Larrañaga A., Blokland M. H. (2022). Insights of Ion Mobility Spectrometry and Its Application on Food Safety and Authenticity: A Review. Anal. Chim. Acta.

[52] May J. C., McLean J. A. (2015). Ion Mobility-Mass Spectrometry: Time-Dispersive Instrumentation. Anal. Chem..

[53] Khajuria R. K., Sharma N., Koul J. L., Verma M. K. (2013). Concentration Dependent Electrospray Ionisation Mass Spectrometry and Tandem Mass Spectrometry (MS/MS) Studies on (E,E)-1-[5-(1,3-Benzodioxol-5yl)-1-Oxo-2,4-Pentadienyl]- Piperidine (Piperine) and Its Analogues. SpringerPlus.

[54] Chen L., Pan H., Zhai G., Luo Q., Li Y., Fang C., Shi F. (2023). Widespread Occurrence of In-source Fragmentation in the Analysis of Natural Compounds by Liquid Chromatography–Electrospray Ionization Mass Spectrometry. Rapid Commun. Mass Spectrom..

[55] Triebel L. J., Wissenbach D., Peters F. T., Wissenbach D. K. (2025). Adduct Formation by Coeluting Endogenous Biomolecules Using ESI-MS. Anal. Bioanal. Chem..

[56] Jiang F., Lu Z., Zhang C., Liu J., Zhu J., Huang M., Zhong G. (2022). Equilibration for Electrospray Ionization Mass Spectrometry in Quantitative Analysis. J. Am. Soc. Mass Spectrom..

[57] Schmid R., Heuckeroth S., Korf A., Smirnov A., Myers O., Dyrlund T. S., Bushuiev R., Murray K. J., Hoffmann N., Lu M., Sarvepalli A., Zhang Z., Fleischauer M., Dührkop K., Wesner M., Hoogstra S. J., Rudt E., Mokshyna O., Brungs C., Ponomarov K., Mutabdžija L., Damiani T., Pudney C. J., Earll M., Helmer P. O., Fallon T. R., Schulze T., Rivas-Ubach A., Bilbao A., Richter H., Nothias L.-F., Wang M., Orešič M., Weng J.-K., Böcker S., Jeibmann A., Hayen H., Karst U., Dorrestein P. C., Petras D., Du X., Pluskal T. (2023). Integrative Analysis of Mul-timodal Mass Spectrometry Data in MZmine 3. Nat. Bio-technol..

[58] Broeckling C. D., Afsar F. A., Neumann S., Ben-Hur A., Prenni J. E. (2014). RAMClust: A Novel Feature Clustering Meth-od Enables Spectral-Matching-Based Annotation for Metabolomics Data. Anal. Chem..

[59] Kuhl C., Tautenhahn R., Böttcher C., Larson T. R., Neumann S. (2012). CAMERA: An Integrated Strategy for Com-pound Spectra Extraction and Annotation of Liquid Chro-matography/Mass Spectrometry Data Sets. Anal. Chem..

[60] Triebel L. J., Wissenbach D., Peters F. T., Wissenbach D. K. (2025). Adduct Formation by Coeluting Endogenous Biomole-cules Using ESI-MS. Anal. Bioanal. Chem..

[61] Jiang F., Lu Z., Zhang C., Liu J., Zhu J., Huang M., Zhong G. (2022). Equilibration for Electrospray Ionization Mass Spectrometry in Quantitative Analysis. J. Am. Soc. Mass Spectrom..

[62] Novoa-del-Toro E. M., Witting M. (2024). Navigating Common Pitfalls in Metabolite Identification and Metabolomics Bio-informatics. Metabolomics.

[63] Bishop L. M., Shen T., Fiehn O. (2023). Improving Quantitative Accuracy in Nontargeted Lipidomics by Evaluating Adduct Formation. Anal. Chem..

