# Correction to “Multi-Laboratory Assessment Reveals Variable Ion Species Profiles in Electrospray Ionization Mass Spectrometer”

**DOI:** 10.1021/jasms.6c00211

**Published:** 2026-06-09

**Authors:** Melanie T. Odenkirk, Jacqueline M. Chaparro, Nathan T. Montgomery, Katrina L. Leaptrot, Stacy D. Sherrod, Jody C. May, Henriëtte van Eekelen, Bert Schipper, Juliana Chaura, Gabriel Esteban Velez, Wimonphan Chathiran, Cole R. Michel, Katrina A. Doenges, Richard Reisdorph, Mari Maeda-Yamamoto, Luis Valdiviez, Uri Keshet, Jeremiah Wells, Scott Mackell, David Bell, Sebastian Tapia Leiva, Simon Partridge, Daniela Rago, Linda Ahonen, Yuki Ito, Margaret L. Read, John C. Evans, Macy J. Gruszczynski, Leokadja Szalach, Stephen C. Boyko, Steven Watkins, Rodrigo Ledesma Amaro, Oliver Fiehn, Nichole Reisdorph, Warangkana Srichamnong, Andres Jaramillo-Botero, Ric C. H. de Vos, Robert D. Hall, John A. McLean, Corey D. Broeckling, Jessica E. Prenni

**Affiliations:** 1 Department of Horticulture and Landscape Architecture, 3447 Colorado State University, Fort Collins, Colorado 80521, United States; 2 Analytical Resources Core, Colorado State University, Fort Collins, Colorado 80521, United States; 3 Department of Chemistry and Center for Innovative Technology, 5718 Vanderbilt University, Nashville, Tennessee 37240, United States; 4 Business Unit Bioscience, 4508 Wageningen University & Research, Wageningen 6708 PB, The Netherlands; 5 iÓMICAS Research Institute, 27965 Pontificia Universidad Javeriana Cali 760031, Colombia; 6 Institute of Nutrition, 26685 Mahidol University, Nakhonpathom 73170, Thailand; 7 Skaggs School of Pharmacy and Pharmaceutical Sciences, 129263 University of Colorado, Aurora, Colorado 80045, United States; 8 Institute of Food Research, National Agriculture and Food Research Organization (NARO), Tsukuba Ibaraki 305-8642, Japan; 9 West Coast Metabolomics Center, Davis, California 95616, United States; 10 Department of Infectious Diseases, Faculty of Medicine, 4615 Imperial College London, London SW7 2AZ, U.K.; 11 Novo Nordisk Foundation Center for Biosustainability, 587234 Danmarks Tekniske Universitet, Kongens Lyngby 2800, Denmark; 12 65828 Shimadzu Corporation, Kyoto 604-8511, Japan; 13 Verso Biosciences, Davis, California 95618, United States

We are requesting a correction of inaccurate author affiliations. Specifically, the correct affiliation for Juliana Chaura, Gabriel Esteban Velez, and Andres Jaramillo-Botero should be iÓMICAS Research Institute, Pontificia Universidad Javeriana, Cali 760031, Colombia

The correct author list and affiliations are shown in the Author Information with this Correction.

