# Supplementary material for: Multi-Laboratory Assessment Reveals Variable Ion Species Profiles in Electrospray Ionization Mass Spectrometry
Source: J Am Soc Mass Spectrom. 2026 May 6;37(6):1376–90. doi: 10.1021/jasms.6c00011 (PMC13750514; doi:10.1021/jasms.6c00011)
Supplement: Supplementary file 1 [file js6c00011_si_001.pdf]

Multi-Laboratory Assessment Reveals Variable Ion Species Profiles in Electrospray Ionization Mass Spectrometry

Supplemental Figures

Melanie T. Odenkirk[1], Jacqueline M. Chaparro[1,2], Nathan T. Montgomery[2], Katrina L. Leaptrot[3], Stacy D. Sherrod[3], Jody C. May[3], Henriëtte van Eekelen[5], Bert Schipper[5], Juliana Chaura[5], Gabriel Esteban Velez[4], Wimonphan Chathiran[6], Cole R. Michel[7], Katrina A. Doenges[7], Richard Reisdorph[7], Mari Maeda-Yamamoto[8], Luis Valdiviez[9], Uri Keshet[9], Jeremiah Wells[9], Scott Mackell[9], David Bell[10], Sebastian Tapia Leiva[10], Simon Partridge[10], Daniela Rago[11], Linda Ahonen[11], Yuki Ito[12], Margaret L. Read[1], J. Christopher Evans[1], Macy J. Gruszczynski[1], Leokadja Szalach[1], Stephen C. Boyko[1], Steven Watkins[13], Rodrigo Ledesma Amaro[10], Oliver Fiehn[9], Nichole Reisdorph[7], Warangkana Srichamnong[6], Andres Jaramillo-Botero[5], Ric C.H. de Vos[5], Robert D. Hall[5], John A. McLean[3], Corey D. Broeckling[2], Jessica E. Prenni[1*]

[1] Department of Horticulture and Landscape Architecture, Colorado State University, Fort Collins, CO 80521, United States

[2] Analytical Resources Core, Colorado State University, Fort Collins, CO 80521, United States

[3] Department of Chemistry and Center for Innovative Technology, Vanderbilt University, Nashville, TN 37240, United States

[4] iÓMICAS Research Institute, Pontificia Universidad Javeriana, Cali 760031, Colombia

[5] Business Unit Bioscience, Wageningen University & Research, 6708 PB Wageningen, the Netherlands

[6] Institute of Nutrition, Mahidol University, Nakhonpathom 73170, Thailand

[7] Skaggs School of Pharmacy and Pharmaceutical Sciences, University of Colorado, Aurora, CO 80045, United States

[8] Institute of Food Research, National Agriculture and Food Research Organization (NARO), Tsukuba Ibaraki 305-8642, Japan

[9] West Coast Metabolomics Center, Davis, CA 95616, USA

[10] Department of Infectious Diseases, Faculty of Medicine, Imperial College London, London SW7 2AZ, United Kingdom

[11] Novo Nordisk Foundation Center for Biosustainability, Danmarks Tekniske Universitet, Kongens Lyngby 2800, Denmark

[12] Shimadzu Corporation, Kyoto 604-8511, Japan

[13] Verso Biosciences, Davis, CA 95618, USA

Corresponding author: jessica.prenni@colostate.edu

KEYWORDS: Electrospray, Ionization, Mass Spectrometry (MS), Metabolomics, Molecule

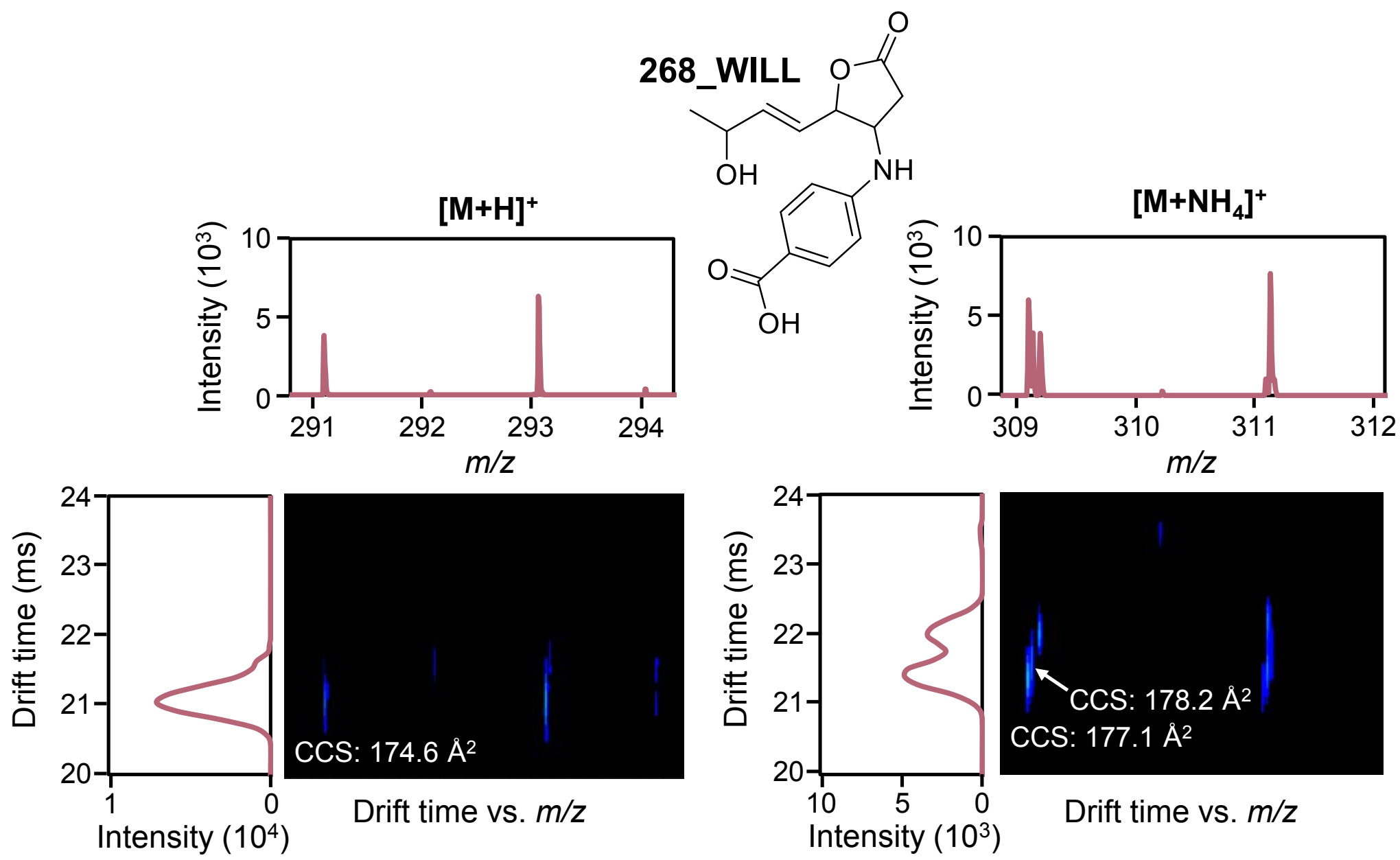

Figure S1. IMS profile data of 268_WILL ammonium and sodium ion species; mass spectrum (top), drift time distributions of the mass range (left), heatmap of the ion mobility data within the mass and drift time spaces (bottom).

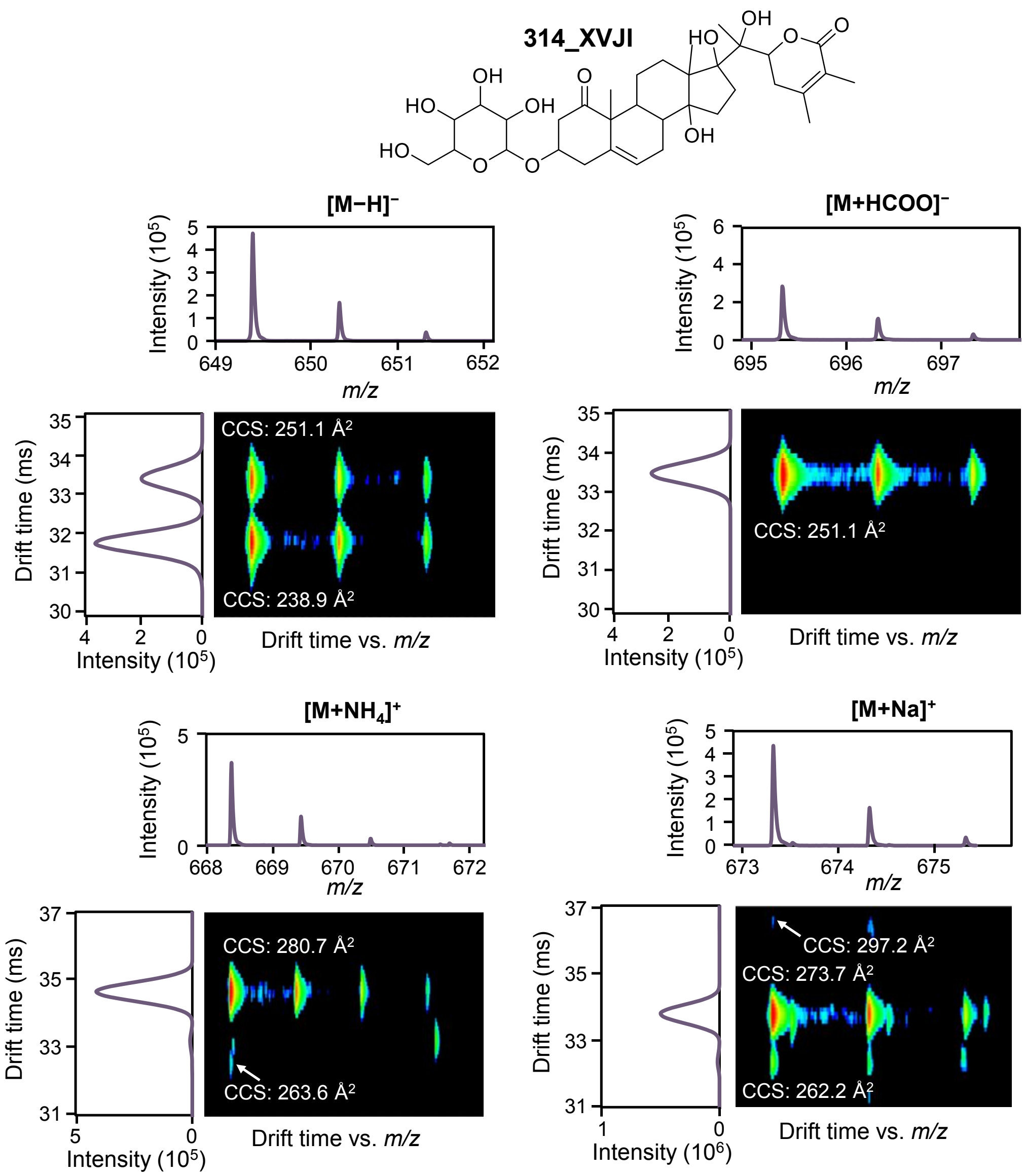

Figure S2. IMS profile data of 314_XVJI deprotonated, formate adduct, ammonium, and sodiated ion species; mass spectrum (top), drift time distributions of the mass range (left), heatmap of the ion mobility data within the mass and drift time spaces (bottom).

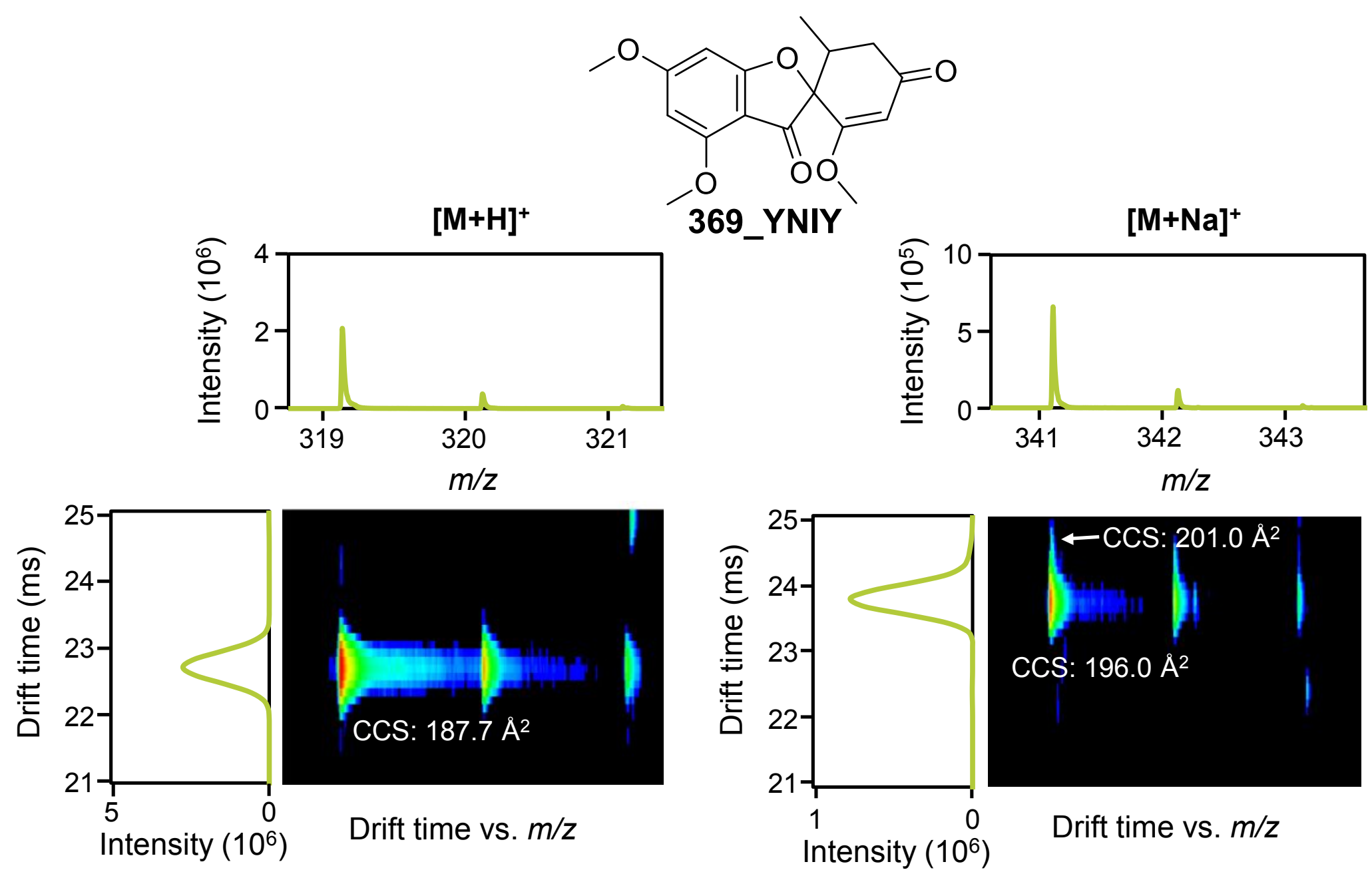

Figure S3. IMS profile data of 369_YNIY protonated and sodiated ion species; mass spectrum (top), drift time distributions of the mass range (left), heatmap of the ion mobility data within the mass and drift time spaces (bottom).

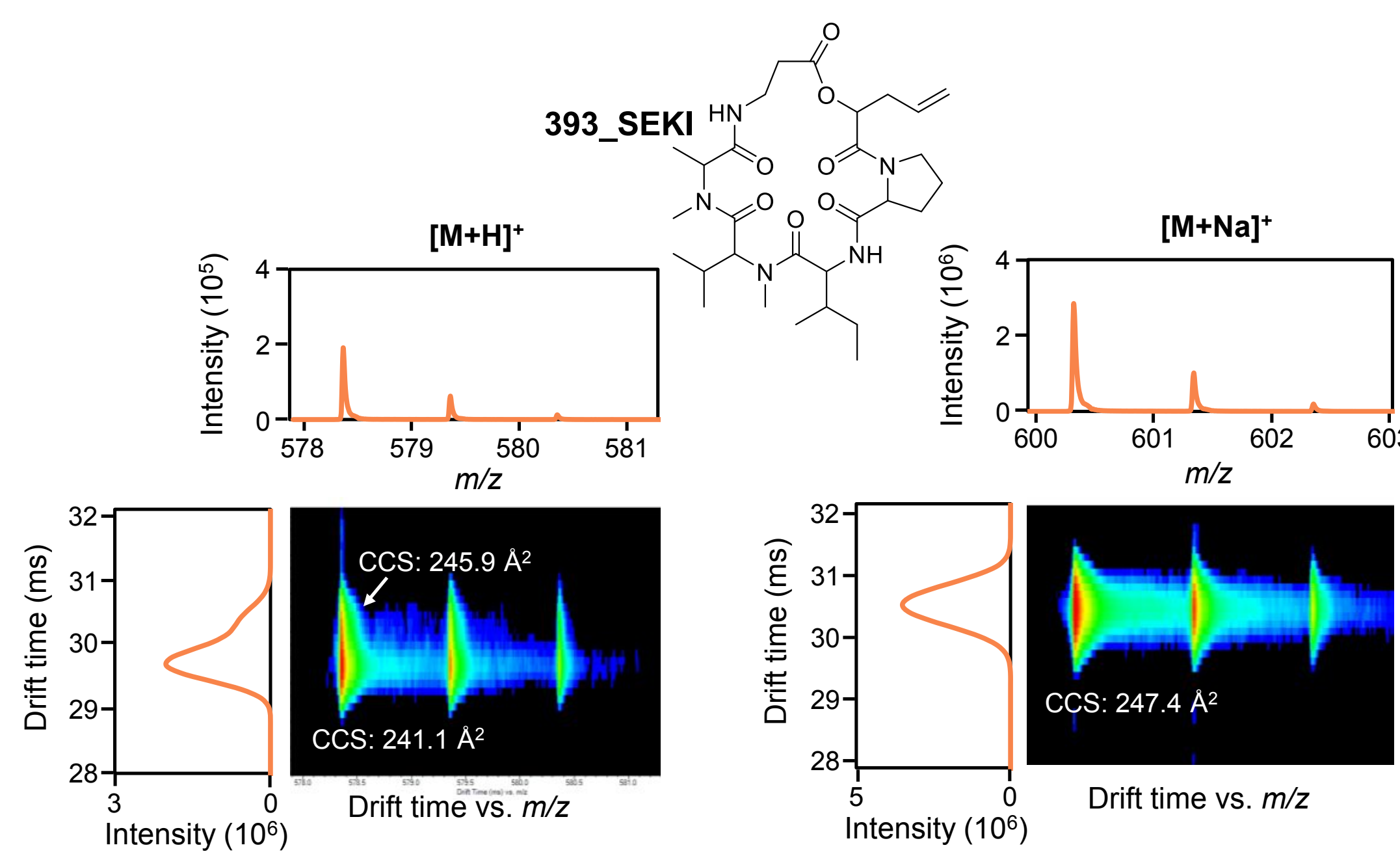

Figure S4. IMS profile data of 393_SEKI protonated and sodiated ion species; mass spectrum (top), drift time distributions of the mass range (left), heatmap of the ion mobility data within the mass and drift time spaces (bottom).

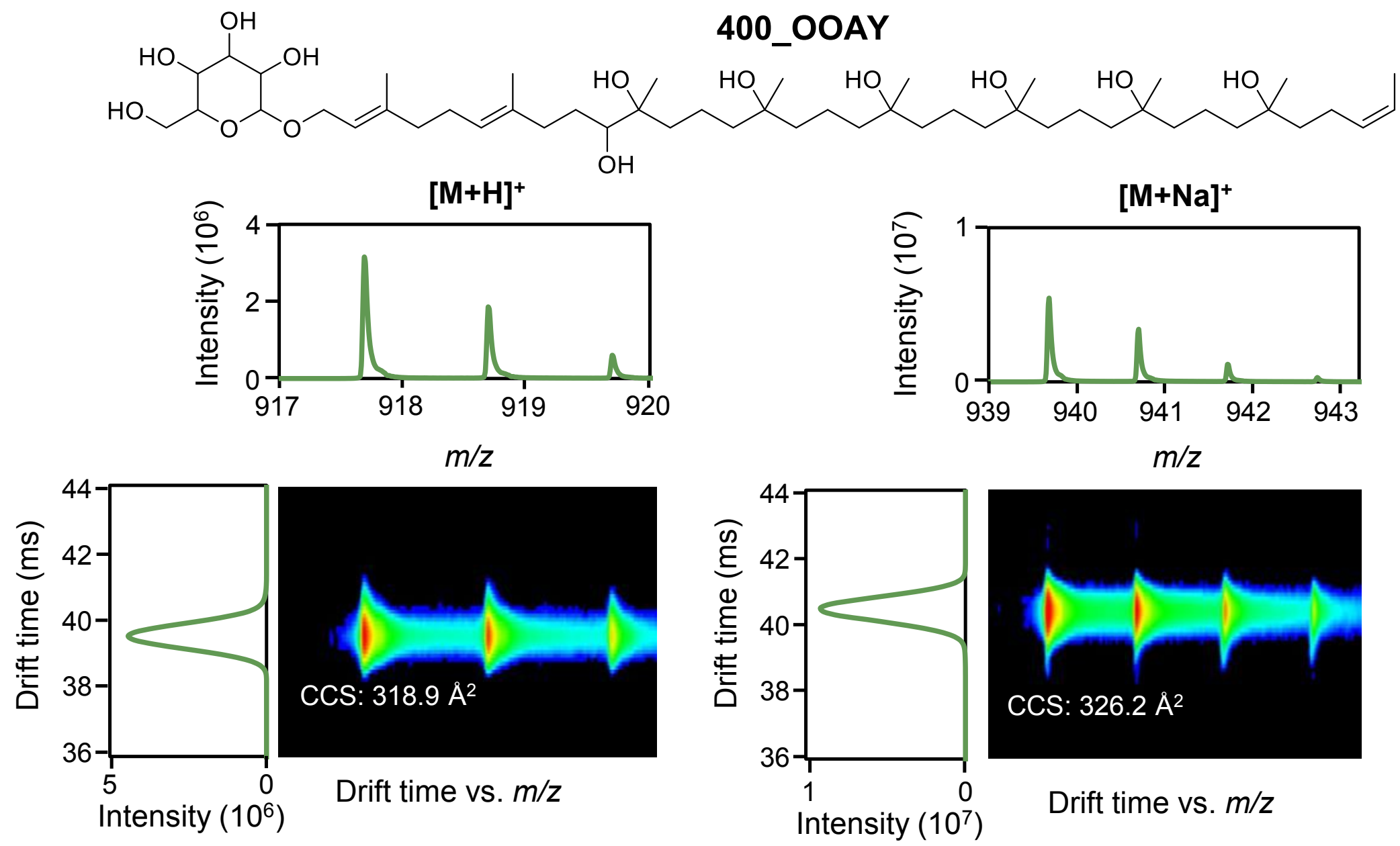

Figure S5. IMS profile data of 400_OOAY protonated and sodiated ion species; mass spectrum (top), drift time distributions of the mass range (left), heatmap of the ion mobility data within the mass and drift time spaces (bottom).

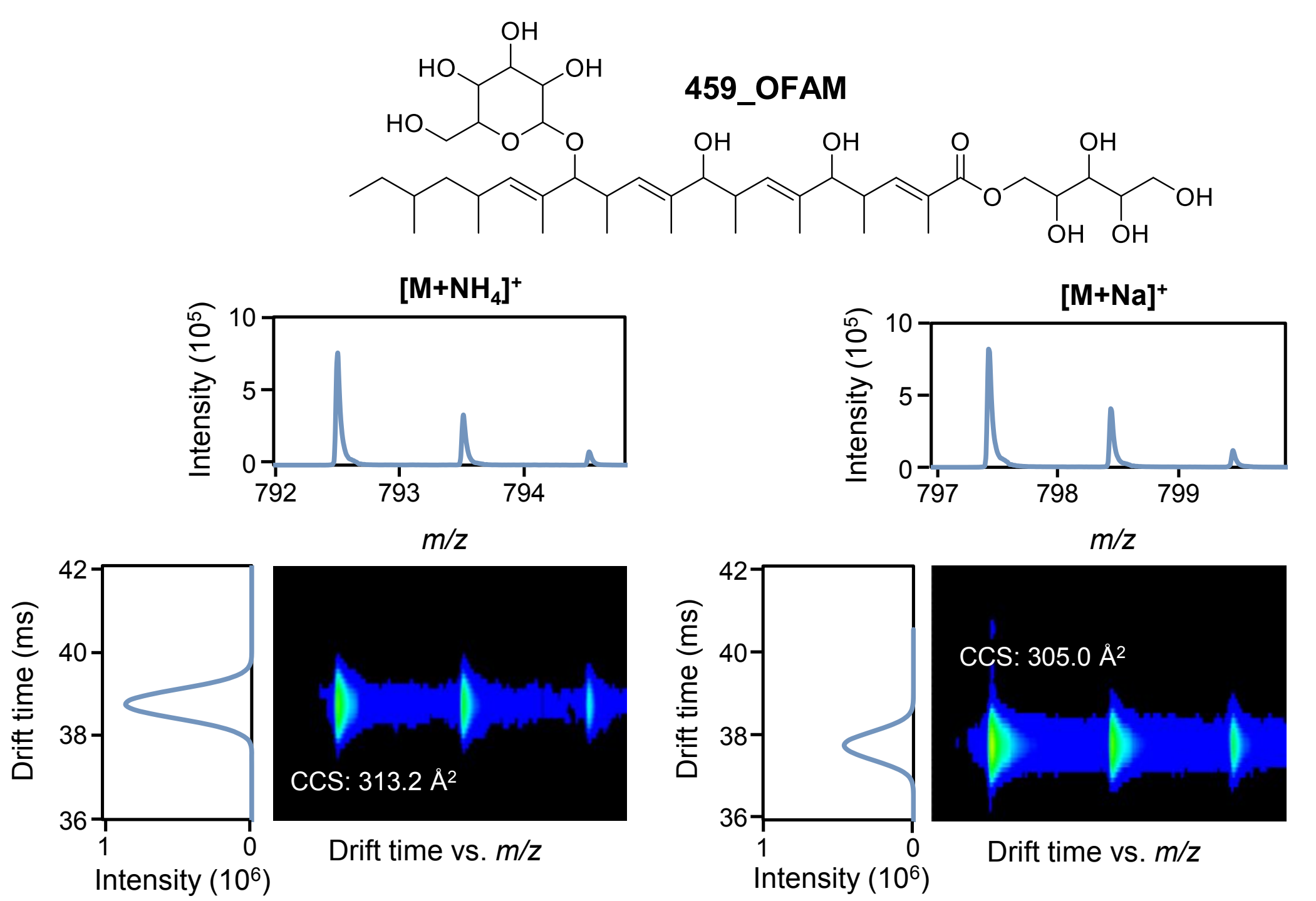

Figure S6. IMS profile data of 459_OFAM ammonium and sodium ion species; mass spectrum (top), drift time distributions of the mass range (left), heatmap of the ion mobility data within the mass and drift time spaces (bottom).

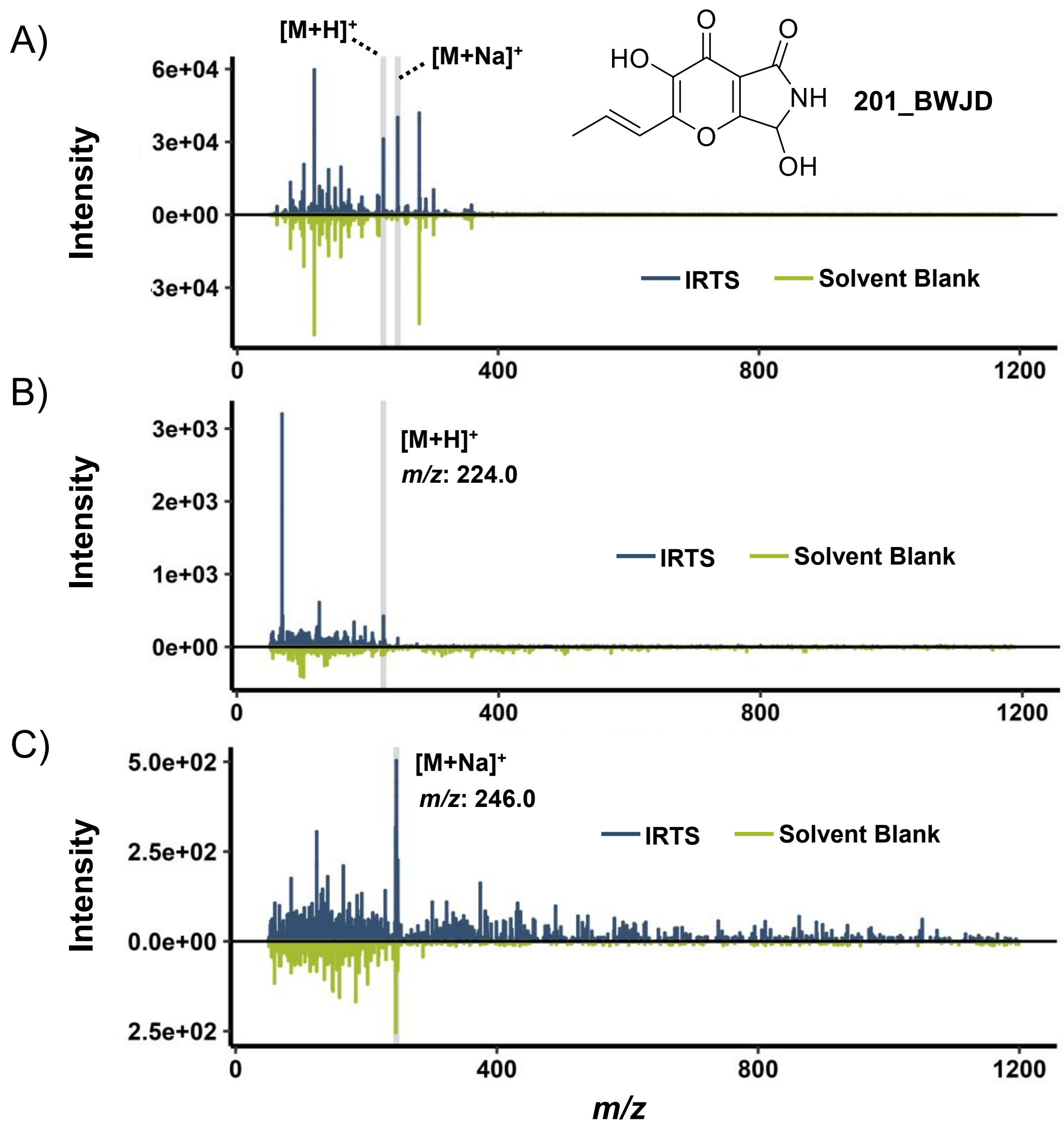

Figure S7. Mass spectra for 201_BWJD IRTS compound. A) Precursor mass spectra for 201_BWJD in IRTS sample (top) and 80:20 MeOH H_2O (inverted spectra) at eluting retention time (3.1 minutes). The grey highlighted regions represent the two ion forms (protonated, $[M+H]^+$ and sodiated, $[M+Na]^+$). B) The DDA spectra for the $[M+H]^+$ ion form (highlighted *m/z* of 224.0) of 201_BWJD in the IRTS sample (top) and 80:20 MeOH H_2O (inverted spectra) at eluting retention time (3.1 minutes). C) The DDA spectra for the $[M+Na]^+$ ion form (highlighted *m/z* of 246.0) of 201_BWJD in the IRTS sample (top) and 80:20 MeOH H_2O (inverted spectra) at eluting retention time (3.1 minutes).

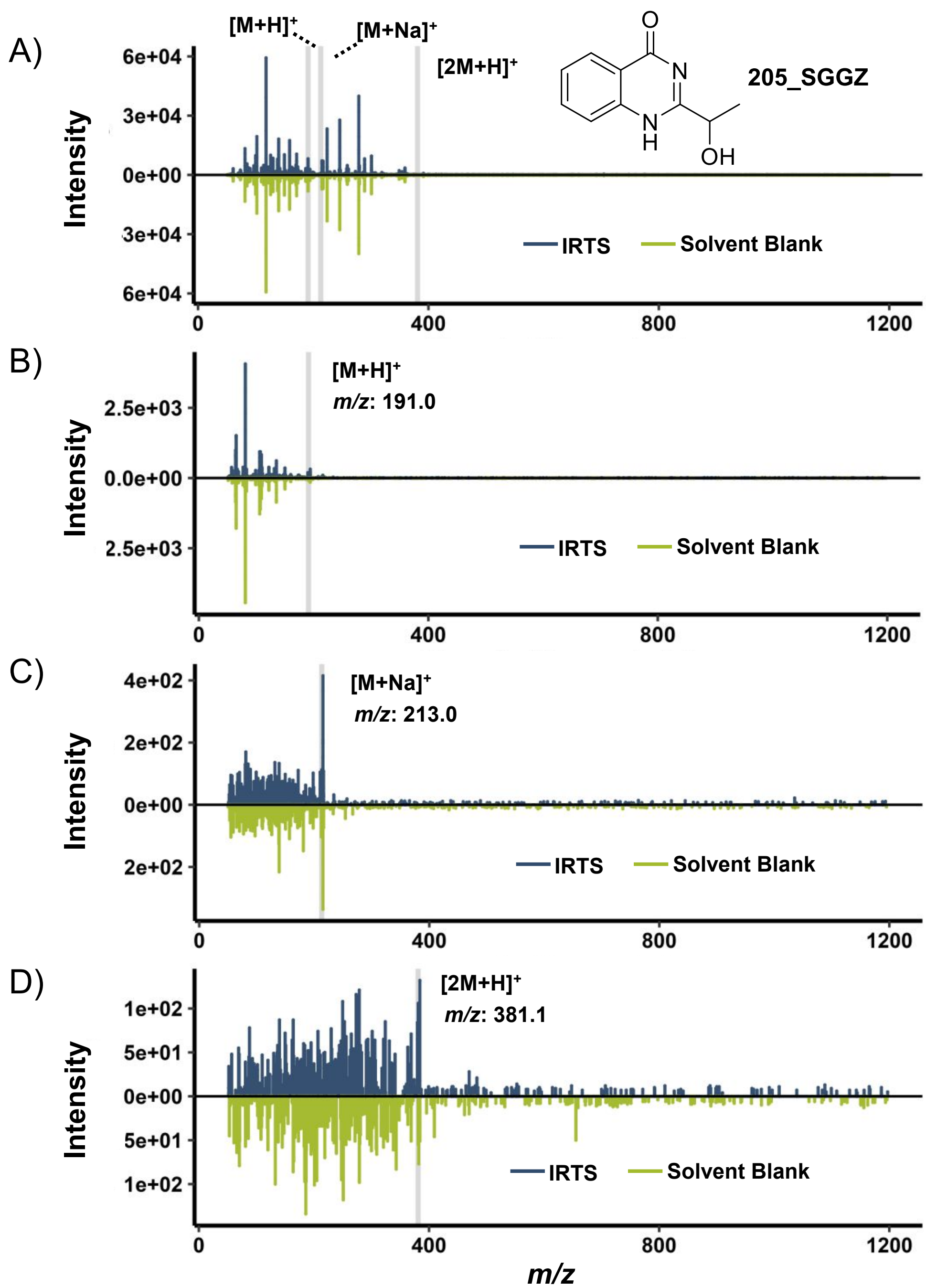

Figure S8. Mass spectra for 205_SGGZ IRTS compound. A) Precursor mass spectra for 205_SGGZ in IRTS sample (top) and 80:20 MeOH H_2O (inverted spectra) at eluting retention time (3.1 minutes). The grey highlighted regions represent the three ion forms ($[M+H]^+$, $[M+Na]^+$, and $[2M+H]^+$). B) The DDA spectra for the $[M+H]^+$ ion form (highlighted *m/z* of 191.0) of 205_SGGZ in the IRTS sample (top) and 80:20 MeOH H_2O (inverted spectra) at eluting retention time (3.1 minutes). C) The DDA spectra for the $[M+Na]^+$ ion form (highlighted *m/z* of 213.0) of 205_SGGZ in the IRTS sample (top) and 80:20 MeOH H_2O (inverted spectra) at eluting retention time (3.1 minutes). D) The DDA spectra for the $[2M+H]^+$ ion form (highlighted *m/z* of 381.1) of 205_SGGZ in the IRTS sample (top) and 80:20 MeOH H_2O (inverted spectra) at eluting retention time (3.1 minutes).

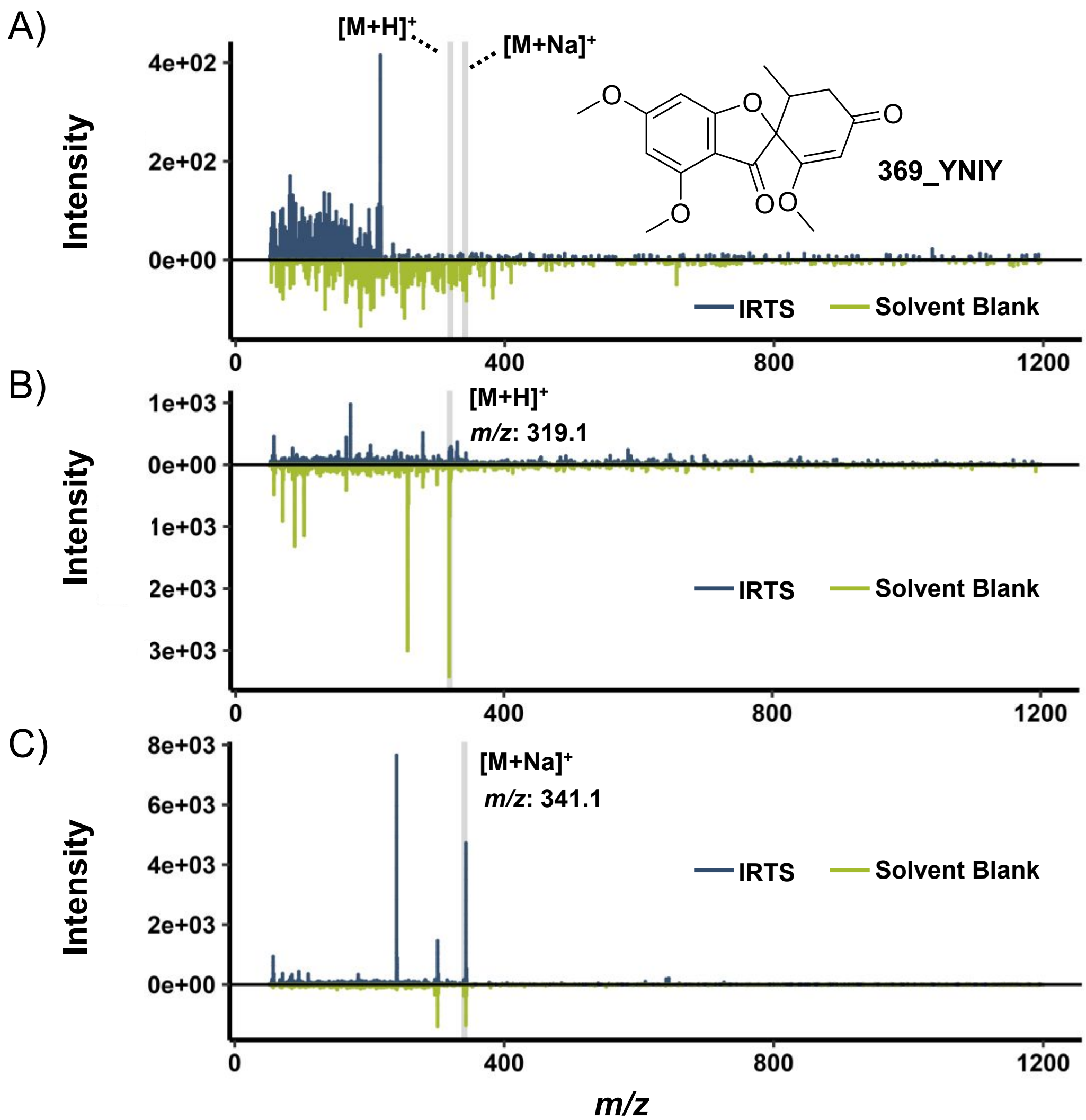

Figure S9. Mass spectra for 369_YNIY IRTS compound. A) Precursor mass spectra for 369_YNIY in IRTS sample (top) and 80:20 MeOH H_2O (inverted spectra) at eluting retention time (5.8 minutes). The grey highlighted regions represent the two ion forms ($[M+H]^+$ and $[M+Na]^+$). B) The DDA spectra for the $[M+H]^+$ ion form (highlighted *m/z* of 319.1) of 369_YNIY in the IRTS sample (top) and 80:20 MeOH H_2O (inverted spectra) at eluting retention time (5.8 minutes). C) The DDA spectra for the $[M+Na]^+$ ion form (highlighted *m/z* of 341.1) of 369_YNIY in the IRTS sample (top) and 80:20 MeOH H_2O (inverted spectra) at eluting retention time (5.8 minutes).

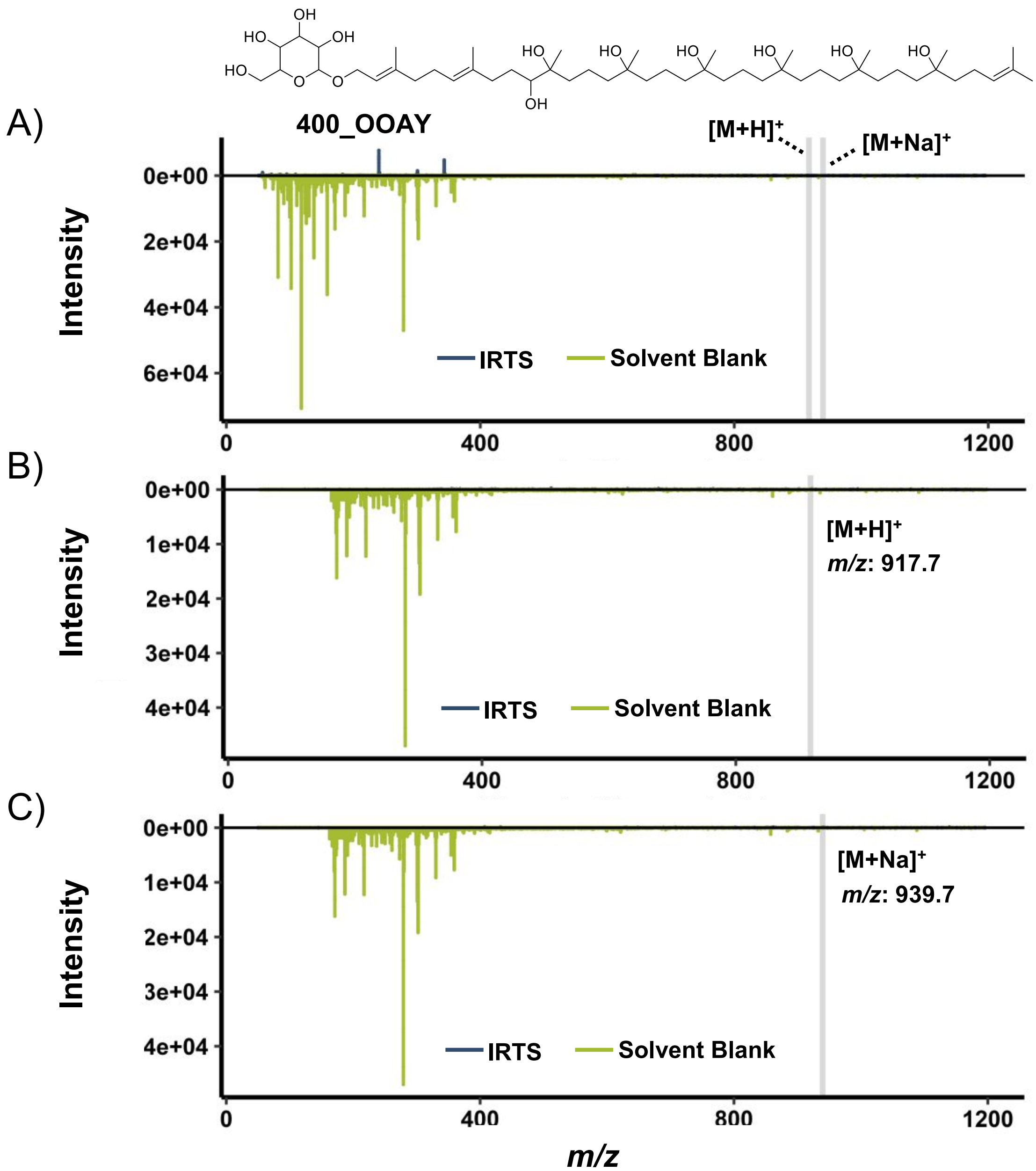

Figure S10. Mass spectra for 400_OOAY IRTS compound. A) Precursor mass spectra for 400_OOAY in IRTS sample (top) and 80:20 MeOH H_2O (inverted spectra) at eluting retention time (6.3 minutes). The grey highlighted regions represent the two ion forms ($[M+H]^+$ and $[M+Na]^+$). B) The DDA spectra for the $[M+H]^+$ ion form (highlighted *m/z* of 917.7) of 400_OOAY in the IRTS sample (top) and 80:20 MeOH H_2O (inverted spectra) at eluting retention time (6.3 minutes). C) The DDA spectra for the $[M+Na]^+$ ion form (highlighted *m/z* of 939.7) of 400_OOAY in the IRTS sample (top) and 80:20 MeOH H_2O (inverted spectra) at eluting retention time (6.3 minutes).

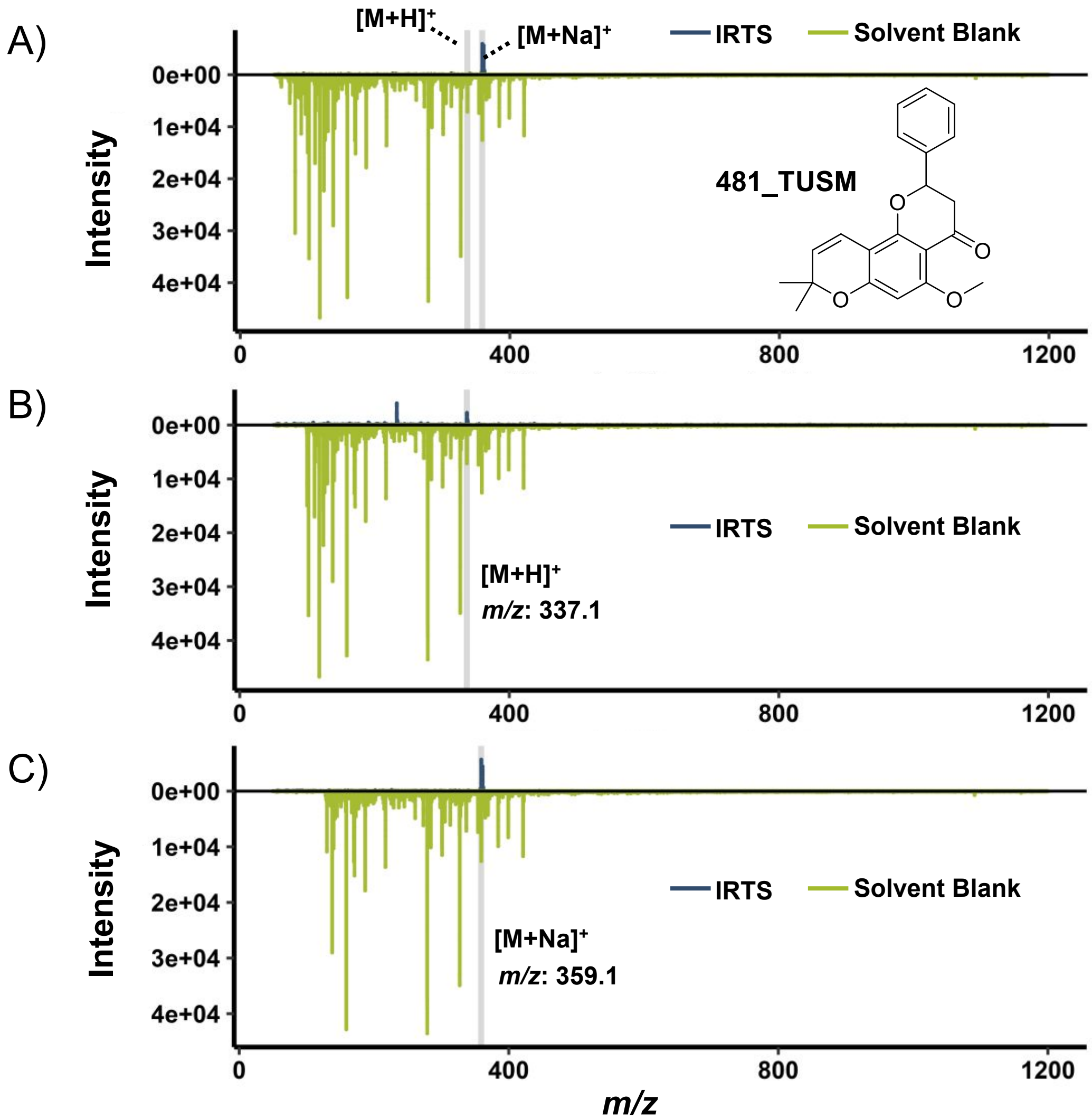

Figure S11. Mass spectra for 481_TUSM IRTS compound. A) Precursor mass spectra for 481_TUSM in IRTS sample (top) and 80:20 MeOH H_2O (inverted spectra) at eluting retention time (7.7 minutes). The grey highlighted regions represent the two ion forms ($[M+H]^+$ and $[M+Na]^+$). B) The DDA spectra for the $[M+H]^+$ ion form (highlighted *m/z* of 337.1) of 481_TUSM in the IRTS sample (top) and 80:20 MeOH H_2O (inverted spectra) at eluting retention time (7.7 minutes). C) The DDA spectra for the $[M+Na]^+$ ion form (highlighted *m/z* of 359.1) of 481_TUSM in the IRTS sample (top) and 80:20 MeOH H_2O (inverted spectra) at eluting retention time (7.7 minutes).

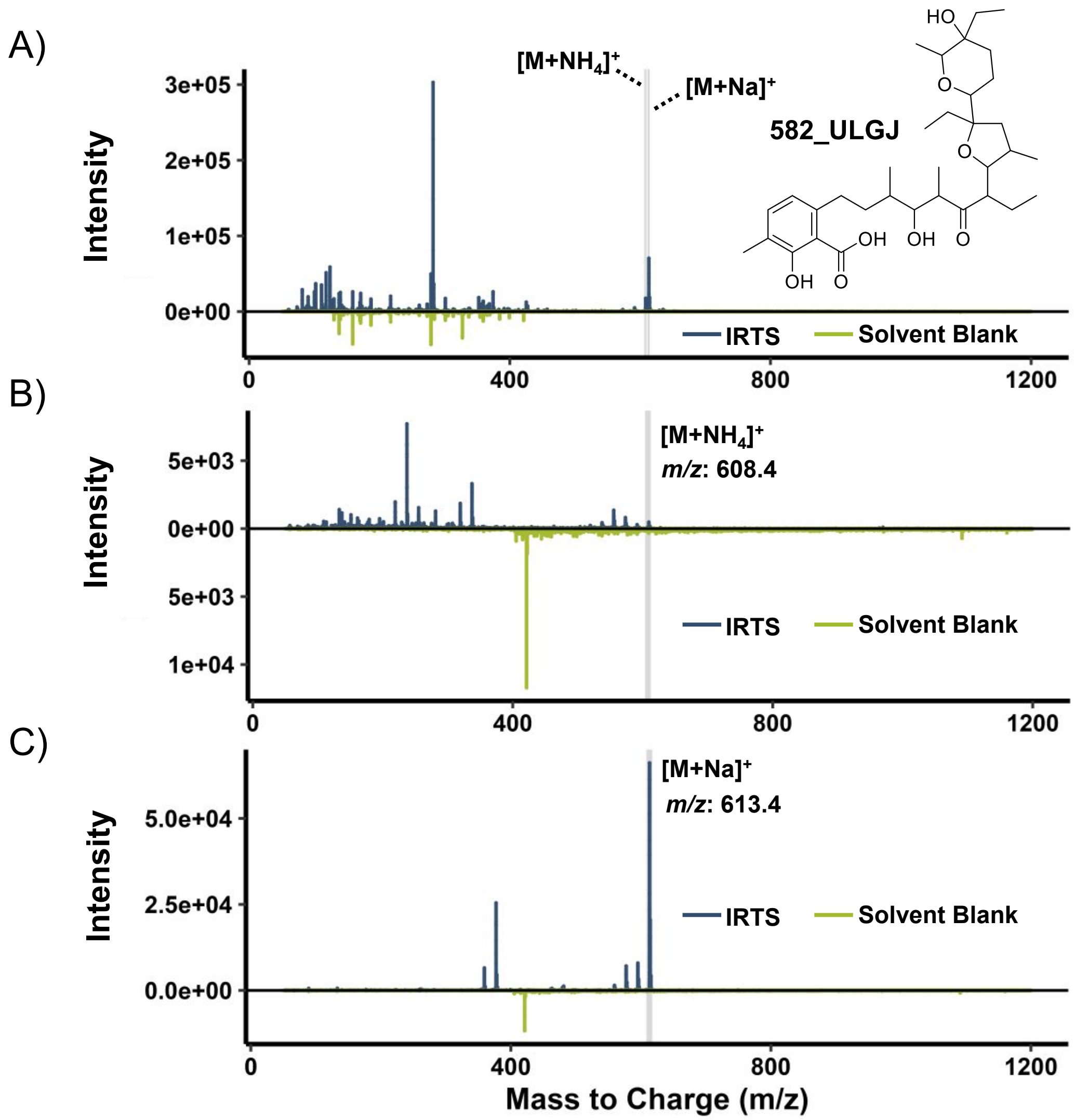

Figure S12. Mass spectra for 582_ULGJ IRTS compound. A) Precursor mass spectra for 582_ULGJ in IRTS sample (top) and 80:20 MeOH H_2O (inverted spectra) at eluting retention time (9.4 minutes). The grey highlighted regions represent the two ion forms ($[M+NH_4]^+$ and $[M+Na]^+$). B) The DDA spectra for the $[M+H]^+$ ion form (highlighted *m/z* of 608.4) of 582_ULGJ in the IRTS sample (top) and 80:20 MeOH H_2O (inverted spectra) at eluting retention time (9.4 minutes). C) The DDA spectra for the $[M+Na]^+$ ion form (highlighted *m/z* of 613.4) of 582_ULGJ in the IRTS sample (top) and 80:20 MeOH H_2O (inverted spectra) at eluting retention time (9.4 minutes).
